# Emerging insights in senescence: pathways from preclinical models to therapeutic innovations

**DOI:** 10.1038/s41514-024-00181-1

**Published:** 2024-11-22

**Authors:** Luke Mansfield, Valentina Ramponi, Kavya Gupta, Thomas Stevenson, Abraham Binoy Mathew, Agian Jeffilano Barinda, Florencia Herbstein, Samir Morsli

**Affiliations:** 1 The Bateson Centre, School of Medicine and Population Health, The University of Sheffield, Western Bank, Sheffield, UK; 2 Cellular Plasticity and Disease Group, Institute for Research in Biomedicine (IRB Barcelona), Barcelona Institute of Science and Technology (BIST), Barcelona, Spain; 3 Department of Cellular and Molecular Biology and Department of Molecular Medicine, The Scripps Research Institute, La Jolla, CA USA; 4 Department of Biomedicine, University of Bergen, Bergen, Norway; 5 Department of Developmental Biology and Genetics, Biological Sciences, Indian Institute of Science, Bangalore, India; 6 Department of Pharmacology and Therapeutics, Faculty of Medicine, Universitas Indonesia, Jakarta, Indonesia; 7 Metabolic, Cardiovascular, and Aging Cluster, Indonesia Medical Education and Research Institute (IMERI), Faculty of Medicine, Universitas Indonesia, Jakarta, Indonesia; 8 Instituto de Investigación en Biomedicina de Buenos Aires (IBioBA) - CONICET - Partner Institute of the Max Planck Society, Buenos Aires, Argentina; 9 Karolinska Institutet, Department of Cell and Molecular Biology, Biomedicum Q6A, Stockholm, Sweden

**Keywords:** Senescence, Cancer, Biomarkers, Experimental organisms, Ageing

## Abstract

Senescence is a crucial hallmark of ageing and a significant contributor to the pathology of age-related disorders. As committee members of the young International Cell Senescence Association (yICSA), we aim to synthesise recent advancements in the identification, characterisation, and therapeutic targeting of senescence for clinical translation. We explore novel molecular techniques that have enhanced our understanding of senescent cell heterogeneity and their roles in tissue regeneration and pathology. Additionally, we delve into in vivo models of senescence, both non-mammalian and mammalian, to highlight tools available for advancing the contextual understanding of in vivo senescence. Furthermore, we discuss innovative diagnostic tools and senotherapeutic approaches, emphasising their potential for clinical application. Future directions of senescence research are explored, underscoring the need for precise, context-specific senescence classification and the integration of advanced technologies such as machine learning, long-read sequencing, and multifunctional senoprobes and senolytics. The dual role of senescence in promoting tissue homoeostasis and contributing to chronic diseases highlights the complexity of targeting these cells for improved clinical outcomes.

## Introduction

Although senescence research has progressed significantly in the last few years, the integration of newly discovered layers of knowledge of this cell state is still a challenge. This has raised new questions and emphasised unanswered ones. Senescence was initially described in 1961 by Hayflick and Moorhead^1^. The in vitro culture of primary human fibroblasts was monitored and their deterioration at around 50 divisions was ascribed to an intrinsic mechanism that led to senescence on a cellular level^1^. Initially perceived solely as a protective mechanism against tumorigenesis^2,3^, senescence is now recognised as a multifaceted phenomenon with diverse roles in tissue homoeostasis, regeneration, and pathology. Therefore, understanding the intricate mechanisms underlying senescence has become paramount for deciphering its roles in health and disease.

Important work has begun to illustrate the interplay between genetic, epigenetic, and environmental factors that influence the regulatory pathways orchestrating senescence induction and maintenance. For example, the identification of key signalling pathways, including the p53/p21^CIP1/WAF1^ and p16^Ink4a^/Rb pathways, has been instrumental in unravelling the molecular triggers driving senescence^4,5^. Moreover, emerging research has shed light on the dynamic nature of senescence, highlighting its plasticity and heterogeneity. The recognition of distinct senescent states, such as stress-induced senescence and replicative senescence, underscores the complexity of the senescent phenotype and its context-dependent regulation^6,7^. Further, recent studies have unveiled the pivotal role of the senescence-associated secretory phenotype (SASP) in orchestrating the crosstalk between senescent cells and the microenvironment, implicating SASP components in tissue remodelling, immune surveillance, and age-related pathologies^8,9^. SASP factors can mediate their effects via autocrine, paracrine, intracrine or even endocrine communication; the same molecule can trigger different signalling pathways with opposite functions^10–12^. These complex characteristics represent a challenge to finding a representative SASP-signature across different senescence models. More than a decade has passed since the first evidence in preclinical models that the elimination of senescent cells alleviates age-associated diseases^13^. However, limitations in reliable senescence biomarkers for tracking senescent cells in vivo or in vitro represent a barrier for the effective translation of senescence research to improving clinical outcomes. As well as senescence biomarkers for diagnostics, the field also requires reliable and reproducible biomarkers for effective senolysis in vivo^14^.

While senescence shares common features, recent advancements in spatiotemporal single-cell analyses are revealing a surprising level of diversity. This diversity depends on the cell type, trigger, and the dynamic state the cells transition through temporally^15^. Most senolytic discoveries have been made in primary cultures or cell lines where senescence is induced in vitro. Although candidates are tested in vivo models, questions arise about the forced conditions under which in vitro senescence is triggered and how representative these conditions are of natural vulnerabilities. The field is debating whether senolytic research should focus on identifying universal senolytics or those targeted to specific tissues or diseases, given the heterogeneous nature of senescence^16^. Additionally, due to this diversity one questions whether a universal senescence biomarker exists, or whether we must instead identify context-specific signatures of senescence.

It is widely acknowledged that senescence plays a pivotal role in the physiology and pathology of many diseases, at least pre-clinically. The field has been rapidly advancing in research aimed at understanding how to effectively manipulate these cells. However, as our understanding of the physiological role of senescence remains limited, concerns persist regarding the potential consequences of completely eliminating or neutralising these cells.

Senescence is a cellular phenomenon that is evolutionarily conserved among different species^17^. As a consequence, the translation into humans could be potentially considered faster or more accurate in comparison with diseases or biological processes that are not represented well in model organisms. Consideration must be given to the limitations of animal models, particularly regarding the understudied long-term impacts of eliminating senescent cells and the varied responses observed across sexes^18^.

In this Review, we describe the latest advances concerning the characterisation, identification and clinical impact of senescent cells, alongside ongoing clinical trials and innovative aspects of senotherapy. We delve into the future directions of the field and explore our predictions regarding emerging senescence research.

## Novel characterisation of senescent cells

In the evolving landscape of biogerontology, the study of senescence has emerged as a cornerstone of ageing research. Here we investigate the recent updates to the identification and characterisation of senescent cells. Advancements in molecular biology techniques, both in vivo and in vitro, have provided unprecedented insights into the senescent state where advanced genomic and proteomic profiling enables in-depth characterisation. Furthermore, a critical evaluation of the emerging characterisations of senescent cell heterogeneity is explored, unravelling the complex roles these cells play in various ageing-related pathologies and tissue regeneration. Through a detailed review of recent literature, this section aims to highlight how these advancements are not only enhancing our understanding of the heterogeneity of cellular ageing but also paving the way for novel anti-ageing therapies.

## Conventional senescence characterisation

A pertinent issue in the study of senescent cells has, for a long time, been their accurate characterisation. The classical characteristics most discussed are lysosomal Senescence-Associated β-Galactosidase (SA-β-Gal) activity and the p16^Ink4a^/RB and p53/p21^CIP1/WAF1^ pathways^19,20^. SA-β-Gal is a lysosomal hydrolase encoded by the gene *Glb1* and its activity is detected in senescence at non-optimal pH (pH = 6). p53 has been well known to be upregulated during stress conditions. An increase in p53 activity can trigger cell cycle arrest and apoptosis. p53 binds to the p21^CIP1/WAF1^ (from now on p21) promoter leading to an increase in p21 expression and protein levels. p21 binds to all cyclin and cyclin-dependent kinases (CDK) pairs and inhibits them to prevent hyperphosphorylation of RB. Hypophosphorylated RB binds to E2F transcription factors which leads to cell cycle arrest^21,22^. Similar to p21, p16^Ink4a^ (from now on p16) binds to CDK 4/6 which prevents phosphorylation of RB, thus preventing transcription of E2F-dependent genes, preventing cell cycle progression^23^. In the lab, scientists can quantify the number of potentially senescent cells using staining methods for SA-β-Gal or measurement and quantification of p16 and p21 expression^24^. Further, the idea of measuring differential gene expression has been proposed as a way to better quantify senescent cells^25^. However, senescent cell populations are heterogeneous, owing to their characterisation using markers that are not exclusive to senescent cells. For example, SA-β-Gal expression is seen in osteoclasts, neurons, and macrophages regardless of senescent induction methods^26–28^. p21 is regulated by the circadian clock and can be upregulated in quiescent cells during the DNA Damage Response (DDR)^29^. p16 expression is also seen during activation and polarisation of macrophages without senescence induction^27^. Therefore, novel research is examining how to best characterise senescent cells so that their impact on disease state can be better understood.

## Senescence characterisation by sequencing

With the advancement of sequencing and screening technologies, databases for senescent cell characterisation are now being established^15,30,31^. SeneQuest was developed by members of the International Cell Senescence Association (ICSA) in 2019 as a novel database for the identification of genes associated with senescence^32^. The SeneQuest website aimed to create a central hub that compiled information from databases in the published literature on senescence. It is currently on its 6th iteration as of June 2023. CellAge was curated as a database of 279 human genes that drive senescence. This includes a majority of genes that affect replicative senescence, as well as stress-induced senescence and oncogene-induced senescence. Both genes that induce and inhibit senescence are included and are classified according to context. Meta-analyses demonstrated some overlap between senescence subtypes, and CellAge genes are overexpressed with age and correlate with cancer. Mass spectrometry of the soluble proteins from multiple human cell types and senescence inducers was used to create the SASP Atlas^31^. Here the authors identified core, inducer and cell-type-specific SASP signatures that include soluble proteins and Extracellular Vesicles (EVs). When comparing these proteins with those significantly associated with age in human plasma, there was an enrichment for proteins from the SASP atlas.

A geneset called SENCAN was created specifically for the context of senescence in cancer, the expression profiles of which are also available online at the Cancer SENESCopedia^33^. This utilised 13 cancer cell lines from four cancer types, treated with either an aurora-kinase A inhibitor, Alisertib, or the topoisomerase II inhibitor, etoposide. Senescent heterogeneity was clear here. Differentially expressed genes changed over time, the SASP was heterogeneous across cell types and susceptibility to the senolytic ABT-263 (Navitoclax) was varied according to context. To more deeply understand the genetic characteristics of senescence, Saul et al. developed a novel geneset designed to highlight senescent cells in heterogeneous populations, entitled SenMayo^15^. The researchers generated a list of 125 genes indicated to be enriched in senescent cells from either human or mouse datasets. The authors excluded p16^INK4A^ and p21^CIP1^ to later validate the data set but included SASP factors, transmembrane, and intracellular proteins. Using mRNA-seq data from bone marrow samples taken from elderly patients the team found that their SenMayo geneset was significantly enriched. Further, testing the SenMayo geneset against publicly available mRNA-seq datasets from young and old mice brains demonstrated significant enrichment with brain age. Finally, Saul et al. used the geneset to assess senescent cell burden in samples taken from individuals before and after a senolytic course of Dasatinib and Quercetin. The researchers found a significant reduction in the expression of genes in the SenMayo geneset in sequencing data taken from the study participants’ adipose tissue biopsies. Here, we see early evidence that the SenMayo geneset may form the basis for future assessment of senescent cell burden in patients.

Cell-type-specific gene sets are being established for more specialised senescence assessment. SenOmic is an online database of senescent human fibroblasts, collating 119 transcriptomic datasets that encompass different senescence types such as oncogene-, DNA damage-, replicative- and bystander-induced senescence^34^ and timeframes post senescence induction. EndoSen is a newly published gene signature for identifying senescence in endothelial cells^35^. Human cord blood-derived primary endothelial colony-forming cells (ECFCs) from 3 donors were induced to either replicative, drug-induced or radiation-induced senescence and sequenced for altered gene regulation. 79 upregulated and 209 down-regulated genes formed EndoSen. The geneset was consistent when instead using human retinal microvascular endothelial cells (HRMECs) and primary retinal endothelial cells from aged mice. EndoSen had an enhanced Normalised Enrichment Score (NES) compared to aforementioned datasets such as SenMayo and CellAge when assessing senescence in endothelial cells, but not in fibroblasts. These efforts demonstrate where focusing on nuance rather than universality may provide novel understanding of the senescence phenotype.

At the single-cell level, a machine learning programme for the identification of senescent cells (SenCID) has been created^36^. This was trained on 52 studies including 602 samples, 57 cell lines and 30 cell types. Six unique senescence identities (SIDs) were identified that were able to distinguish senescence from other states in nearly all cell types. SID scores increased with age and age-associated diseases and could be used in high-throughput single-cell CRISPR screens to identify genome-wide senescence trigger and suppressor genes. Two preprints have also recently emerged for the classification of senescence data from single-cell analysis, with potential for in vivo assessment^37,38^. Hughes et al. demonstrated SenPred, a machine learning pipeline made from 2D and 3D fibroblast models of senescence, the latter more accurately predicts senescence in vivo from published datasets^38^. Sanborn et al. developed SenePy with a mouse single-cell ageing atlas from the Tabula Muris Consortium. This resource comprises over 300,000 cells from 19 tissues across the mouse lifespan up to 30 months of age, as well as human data from 7 studies comprising 1,600,000 cells from 37 tissues across the human lifespan up to 92 years of age. The SenePy signatures shared some common stress responses and inflammatory pathways and elevated signatures with the disease. However, the senescence profiles were extremely heterogeneous and highlighted the importance of these new technologies for the next generation of senescence research. These approaches for senescence assessment at the single-cell level and in vivo will potentially help to uncover senescent subtypes, such as those detrimental to a specific disease context with a unique vulnerability to be exploited therapeutically.

While the majority of aforementioned novel senescence signatures are transcriptomic, it would be key to identify senescence characteristics using alternative omics. Mapping senescence signatures into spatial omics would better define their relationship with proximal and distal cell types, as well as their association with physiology and pathology. The aforementioned SASP atlas well describes the proteome of the SASP^31^, but deeper proteomics into senescent cells, including metabolic signatures would prove beneficial for effective senescence characterisation. A recent preprint describing a mass-spectrometry-based screen for proteomic and metabolomic changes in senescence demonstrates metabolic pathways altered during senescence and the differences seen according to the senescence induction method^39^. Additionally, proteomics screening has been used to assess senescence in a more cell-type-specific manner^40^. Assessment of somatic mutations in senescent cells by single-cell whole genome sequencing in early or late passage human fibroblasts also demonstrated an increase in aneuploidies in senescent cells^41^. Epigenetic alterations have been identified in senescence, and are dependent on the induction method, though epigenetic signatures for in vivo identification have been limited^42–47^. These studies demonstrate where strides can be made in senescence characterisation using epigenomics, proteomics, genomics and metabolomics.

## Senescence heterogeneity

With technological advancements in recent years, it has become more apparent than ever that biology is inherently heterogeneous. In this regard, it is now well understood that the senescent phenotype is an example of this. To disentangle senescence heterogeneity, we must first understand the specific contexts of senescence, such as in different organs and diseases, and highlight where the variabilities lie.

As an example, the in-depth characterisation of senescence in the brain is a pertinent area of research with noticeable heterogeneity. Research by, Bussian et al. ^48^, observed the impact of senescent cell accumulation on the onset of Alzheimer’s disease. Using the *MAPT-P301S-PS19* mouse model to study neurofibrillary tangles (NFTs), the group observed that the accumulation of *p16+* astrocytes and microglia were causally linked to the deposition of NFTs and associated loss of cognition. Further, research by Matsudaira et al. demonstrated an accumulation of p16-positive microglia in the brains of aged (18-months-old) mice that are associated with SASP genes Il1b and Cxcl10^49^. Additionally, single-cell RNA-sequencing showed *p16*-positive cells derived from the aged mice corpus callosum were identified as damage-associated microglia (DAM), known to exacerbate neuronal inflammation^50^. Therefore, data suggests senescence accumulation in the aged brain may contribute to the onset of age-related neuropathies, providing a promising avenue of research aimed at delaying age-related neurological diseases^48^.

While senescent microglia seem causal to age-related brain disorders, senescence in neurons is controversial. While neurons cultured for a long time do show some indicators of senescence, including DNA damage, the secretion of SASP components such as IL-6, and mitochondrial dysfunction, they lack others, such as telomere attrition^51–53^. In a study conducted by Palmer et al. increased *P16* staining along with other senescence markers was observed in myenteric neurons of the ascending colon in older individuals suggesting a region-dependent, post-mitotic cellular senescence-like activity, perhaps associated with the ageing of enteric neurons within the colon^54^. This post-mitotic senescence-like phenotype is also observed in the brains of SARS-CoV-2 affected individuals showing accelerated signs of ageing- suggesting heterogeneity in the mode of induction as well^55^. Also, unlike the role of senescent cells in wound healing, tumour suppression, and embryonic development, the physiological ramifications of senescence in post-mitotic cells remain poorly understood^56^.

While there is ample literature on the detrimental effects of senescent cells, we must also examine the other roles of senescent cells in wound healing and development. For example, Yao et al. examined the role of senescent cells in lung development in newborn mice (postnatal day 0–7)^57^. It was observed that mice exposed to hyperoxia developed senescent cells that demonstrated an increase in pro-inflammatory marker expression (such as *Il-1α*, *Il-1β*, *Cxcl2*, *Cxcl12*, and *Tnfrsf1b*) indicating an increase in senescent cell burden even in young mice as a result of the damage caused by hyperoxia. However, when the group observed senescent cells in neonatal mice with normoxia the researchers noted a population of senescent cells that decreased over time (postnatal day 0–7). Importantly, it was reported that this population of senescent cells did not show signs of DNA damage such as increase ɣH2ax, and did not show an increase in the pro-inflammatory markers *IL-1α*, *IL-1β*, *Cxcl2*, *Cxcl12*, and *Tnfrsf1b*. The researchers concluded that these early senescent cells are essential for lung development. This highlights both the importance of senescence in the neonatal development of mice and the heterogeneity of the senescent cell population in the lung, drawing attention to the need of in-depth characterisation of senescent cells for an accurate study of their role in organismal health.

These points are further supported by *Reyes* et al. where they investigated *p16*-expressing fibroblasts in the lung^58^. The researchers found a group of fibroblasts in the lung that were *p16-positive* in newborn mice. However, the group also found that these fibroblasts persist within the stem cell niche, promoting the repair of barrier injury at the epithelium^58^. This indicates that there may be populations of cells, defined as senescent, that are integral to the wound healing process, being ablated with first-generation senolytics. With the technological advancements of today, we must move away from the simplistic classification of senescence to avoid this. As a further example of this, *Chandra* et al. performed a comparison of the effects of ablating either *P21* or *p16*-positive cells^59^. Initially the research group induced a senescence phenotype via radiation-induced osteoporosis. When selectively removing *p21* cells the team found a reduction in SASP markers, a reduction in the number of senescent osteocytes, and that bone loss and bone marrow adiposity were reduced indicating that the negative impact of the radiation-induced senescence had been ameliorated. However, the researchers did not find the same effects upon the removal of *p16* cells. Additionally, *p16* cells are required for mouse liver health^60^. Following ablation of *p16* cells in the livers of 12-month-old mice, removal did not lead to the replacement of lost cells by the division of healthy neighbours, but instead by fibrosis, overall leading to a detrimental effect on mouse health^60^. Considering that a high percentage of the p16 cells were reported to be macrophages and liver sinusoidal endothelial cells (LSECS), which are integral to liver immune function, it is clear why p16 ablation will lead to a detrimental effect on organismal health in this context^61^. These demonstrate the nuance required when targeting senescent cells for a specific age-associated disease.

It is evident that the blanket removal of senescent cells based on classical markers may not be the best approach to alleviating the negative effects of senescence. Further studies should investigate the heterogeneous nature of cells marked by the p16. While p16 and p21 are undoubtedly important markers of senescence, identifying additional in vivo markers that distinguish positive and negative aspects of senescence is needed.

A better understanding of senescence heterogeneity will enable advances in ameliorating the negative impact of senescence on organismal health. One method that can provide high-quality data on senescence heterogeneity is single-cell RNA sequencing. Saul et al. conducted a meta-analysis of single-cell RNA-sequencing datasets from both humans and mice in order to investigate the roles of *p21* and *p16*^62,63^. The group found that while both *p21* and *p16* are conserved across tissues and organisms in the induction of senescence, it is not the rule that they are both involved in the induction of senescence. Further, the evidence suggests that cells expressing *p21* and *p16* constituted subpopulations with distinct SASP compositions. What is more, not only have distinct subpopulations of senescence been identified in different tissues, but they have also been identified in senescent populations derived from clonal cell lines. For example, Evans et al. investigated the single-cell transcriptomic landscape of oncogene-induced, DNA-damage induced and replicatively senescent IMR90 fibroblasts^64^ and showed that overall, there was a clear senescence signature compared to proliferating controls, but there was marked heterogeneity within the senescence populations, even in ‘central’ senescence genes p21 & p16. Additionally, there was altered sensitivity to the senolytic Navitoclax.

The evidence that senescence is heterogeneous in nature, as with many biological processes, is clear. However, due to the extensive literature describing senescence ablation leading to relevant improvements in treating many age-related disorders, heterogeneity does not inherently limit the potential therapeutic benefit of modulating senescent cells in vivo. Rather, it underpins the need for further characterisation of this will contribute to the development of more effective senotherapies with increased specificity and sensitivity.

## in vivo senescence in non-mammalian model organisms

**Fig. 1:**
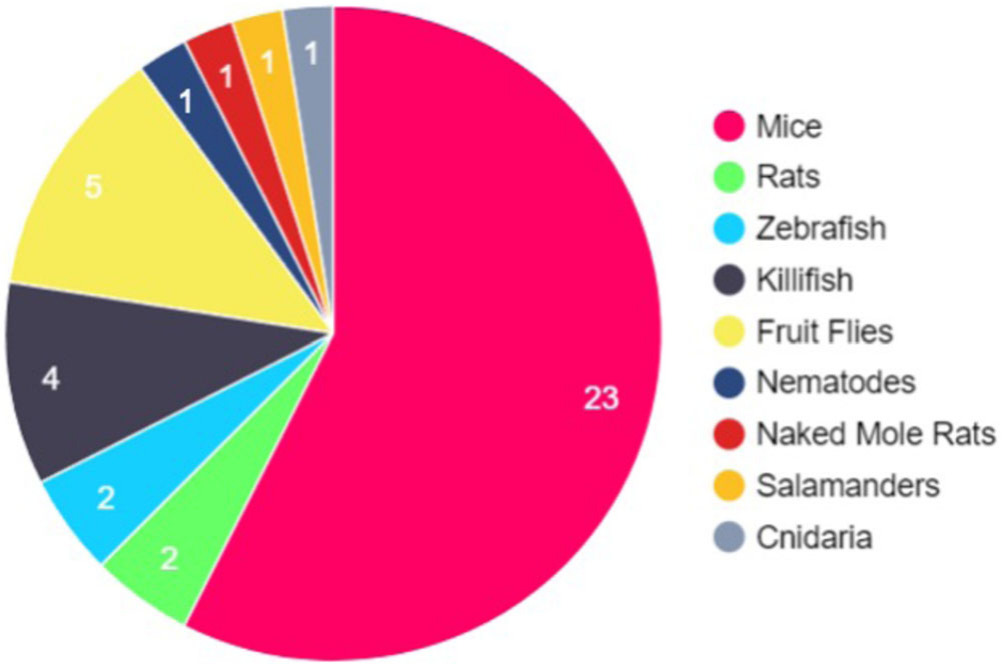
Common model organisms used to study senescence including genetically altered variants. The study of senescence employs many different model organisms. Observing the number of different model organisms, we can see that those used are heavily weighted towards mice with 23 different genetically engineered strains used to study senescence and senotherapies. Mice are genetically manipulable and are considered the gold standard for most animal studies which has led to them being the most common animal used to study senescence. However, the other model organisms listed have their own advantages such as the ease of imaging different tissues while alive in Nematodes, or the short life cycle of Killifish.

Studying the fundamental and interconnecting mechanisms of senescence in vivo is essential for the development of therapeutic interventions for senescence-driven diseases, to identify novel biomarkers of such diseases, and to investigate the developmental origins and purposes of senescent cells. Cultured cell lines do not capture the physiological complexity of an entire organism, and thus key disease-relevant information on the roles of senescent cells between organs and systems cannot be obtained. To date, many in vivo studies of ageing and senescence have been conducted in mice (*Mus muscularis)*. However, in recent years many alternative model organisms of senescence have emerged. These include cnidaria (*H. symbiolongicarpus)*, fruit flies (*Drosophila melanogaster)*, nematodes (*Caenorhabditis elegans)*, killifish (*Nothobranchius furzeri), zebrafish (Danio rerio)*, axolotls (*Ambystoma mexicanum)*, newts (*Notophthalmus viridescens)*, frogs (*Xenopus laevis*) and Rats (*Rattus norvegicus*) (Fig. 1). Model organisms used in senescence research and their transgenic reporters are highlighted in Table 1. By utilising these model organisms, studies have uncovered senescent cells and tissues across several organ systems, and elucidated essential roles of senescent cells in embryonic development and tissue regeneration, highlighting new insights into the evolutionary origins of cellular senescence^7,65–72^ We will explore these advancements here.

**Table 1:** Model organisms for the study of senescence

Model organism	Features	Senescence reporter	Ref.
cnidaria (*H. symbiolongicarpus)*	Highly regenerative	*Cdki1::GFP*^*CAAX*^	^82^
	Translucent		
Nematodes *(Caenorhabditis elegans)*	Short-lived	None currently available	
	Easily maintained in the laboratory		
	Transparent body for anatomical observation		
	Genetically manipulable		
	Aged C. elegans display a decline in anatomical and functional features		
	High usability for throughput screening experiments		
Fruit Fly *(Drosophila melanogaster)*	Short-lived	TRE-dsRed	^81^
	Genetically manipulable		
	Low cost of rearing and housing		
	Ease of generating large populations		
	A large collection of readily available genetic tools		
	Tissues are equivalent to many of those found in mammals		
African Turquoise Killifish *(N. furzeri)*	One of the shortest-living vertebrates	p21-GFP	^84^
	Reach sexual maturity within four weeks		
	Similarity to mammals including humans		
Zebrafish *(Danio rerio)*	Inexpensive and high fecundity	p21-GFP	^68^
	Senescence and ageing in a manner comparable to humans		
	Genetically manipulatable		
Salamanders *(Notophthalmus viridescens)*	Ability of extensive regeneration	None currently available	
	Various technologies are available for gene delivery		
	Tools for germline transgenesis		
	Many tissues are optically transparent		
	Highly suited for live imaging		
Axolotl *(Ambystoma mexicanum)*	Highly regenerative	None currently available	
Naked Mole rats *(Heterocephalus glaber)*	Longest-lived rodent	None currently available	
	High tolerance to low levels of oxygen		
	Good model of cellular rejuvenation		
	Resistant to cancer and neurodegenerative diseases		
Rats *(Rattus norvegicus)*	Mammalian model with similarities to human physiology	None currently available	
	Large size allows easier assessment of smaller organs		
Mice *(Mus muscularis)*	Mammalian model with similarities to human physiology.	INK-ATTAC	^13^
		p16-3MR	^70^
	Genetically modifiable.	p16-Cre^ERT2^-tdTomato	^162^
		INKBRITE	^58^
	Shorter lifespan.	p16-FDR	^163^
		p16tdTom	^161^
	Well-described disease models of senescence.	p16 luciferase	^159^
		p16-Cre/R26-DTA	^60^
	Extensively researched biology of ageing.	p21-ATTAC	^59^
	Used for preclinical studies.	p21-Cre	^62,165^
		p21-3MR	^166^
	Small size.	p19ARF-DTR	^167^
		Glb1-2A-mCherry	^170^
		Gpnmb-DTR-luciferase	^172^

The development of in vivo tools to study senescence has progressed the field significantly. This table provides a summary of the invertebrate, vertebrate non-mammalian and mammalian model organisms are described, along with their advantages and transgenic senescence reporters developed, where available.

## Invertebrate models of senescence

The nematode worm (*C. elegans)* is a useful model to study ageing, with the advantages of affordability and a high fecundity allowing small effect sizes to be statistically powered in the experimental design. *C. elegans* has been used extensively as an ageing model for lifespan studies because it has around 60% of its genes homologous to those found in humans and a mean lifespan of around 20 days when cultured at 20 °C^73,74^. For example, Venz et al. identified a novel gene in *C. elegans*, *regnase-1*, that was then found to regulate SA-β-Gal staining in human cell culture^75^. This demonstrates the potential use of *C. elegans* as a screening tool for the investigation of modulators of senescence-associated lysosomal phenotypes. Additionally, Wang et al., assessed a library of compounds extracted from plants from the Yunnan Province, China, in *C. elegans* and identified novel senotherapeutic compounds with in vitro efficacy in human lung fibroblasts and in vivo efficacy in mice that were aged or treated with doxorubicin^72^. The use of *C. elegans* in senescence research should also be closely assessed due to their semelparous-like profile of reproduction and death^76^. Therefore, lifespan-extending discoveries in *C. elegans* could be due to delaying their reproduction. Mammals on the other hand are an iteroparous species, and so findings in *C. elegans* should be clarified in this context for effective translation to human health.

The fruit fly (*D. melanogaster)* has been utilised as a model organism in a vast range of genetic studies, making significant contributions to the fields of disease and development^77,78^. Its popularity in many fields is largely owed to a very short generation time, a relatively large number of offspring, ease of genetic manipulation compared to other model organisms such as mice, and a high degree of mechanistic conservation with other organisms, including mammals^67^. Several studies have described the induction of senescence in Drosophila, induced by irradiation^79^, oncogene activation^65^, or by pharmacological means^80^. Recently, senescent glia was described in the aged Drosophila brain^81^. A reporter line for AP1 (TRE-dsRed), a transcription factor associated with senescence and the SASP, is increased with age alongside SA-β-Gal activity and a fly DNA damage marker, γH2Av. AP1+ glia were isolated and shown to express senescence markers with age. It was discovered that neuronal mitochondrial dysfunction triggered the onset of AP1+ glia, and a mild blocking AP1+ activity extended fly lifespan and reduced SA-β-Gal activity by limiting lipid accumulation. This was confirmed in mammalian cells in vitro. The discovery of senescent cells modifying lipid storage in non-senescent cells in flies demonstrates the usefulness of this model system for rapid assessment of the role of in vivo senescence in ageing.

A study on cnidaria (*Hydractinia symbiolongicarpus)* demonstrated the role of senescence in the regeneration of a highly regenerative organism^82^. Following amputation, senescent cells emerge expressing a P21 paralogue (*Cdki1)* and SA-β-Gal activity and induce reprogramming in neighbouring cells to promote full tissue regeneration. A fluorescent reporter for *Cdkni1* was established (*Cdki1::GFP*^*CAAX*^*)* and used for live detection of senescent cells. Both Navitoclax and Rapamycin were able to limit senescence following amputation, similarly knocking out *Cdki1* stopped senescence-associated regeneration. Ectopically increasing senescent cells enhanced regeneration seen following amputation. This was again lost when *Cdki1* was knocked out. Assessing the role of physiological senescence in a highly regenerative model such as cnidaria could identify the evolutionary conservation of physiological senescence in wound healing and regeneration, and identify tools to improve regeneration in higher-order animals with limited regenerative capacities, such as mammals.

## Senescence in non-mammalian vertebrates

In vertebrate models of ageing, the African Turquoise Killifish (*N. furzeri)* is gaining traction^83^. Originating from sub-Saharan Africa, this species of Killifish is one of the shortest-living vertebrates that can be bred in captivity, reaching sexual maturity within four weeks. This has led to it becoming an increasingly favoured model organism, bridging the gap between the slowly maturing vertebrate model organisms and the less orthologous invertebrates. In the context of senescence, a GFP transgenic reporter for p21 has been established in Killifish using CRISPR/Cas9^84^. Recent work has demonstrated that Killifish are amenable to genetic manipulation, and exhibit some of the hallmarks of ageing observed in humans, including the accumulation of senescent cells, mitochondrial dysfunction, and telomere shortening^85,86^. Additionally, D&Q treatment in aged killifish can reduce the senescence burden in the central nervous system and improve neurogenesis following traumatic brain injury^87^. However, the short lifespan that makes the Killifish such an appealing model may also limit its usefulness. Recent research by Moses et al. indicates that the Killifish lifespan is closely linked to germline cell development and that depletion of germline cells leads to increased lifespan as if the Killifish were displaying a semelparous-like reproductive strategy, as is seen in *C. elegans*^88^. Therefore, more work may yet be needed in characterising how Killifish reproduction is linked to lifespan, so that it may be used most effectively in its role as a model organism of human ageing.

In recent years the use of zebrafish (*D. rerio*) to study ageing has gained popularity. Zebrafish have conserved hallmarks of ageing with humans as well as sharing 84% of known human disease-associated genes and 70% of human protein-encoding genes^89^. Zebrafish develop rapidly, are fertilised externally, have high fecundity and are optically translucent in early development to allow the visualisation of cellular processes live in a whole organism context^90,91^. Genetic alterations have also reduced zebrafish pigmentation to allow visualisation of internal organs even at adult stages, such as the *casper* mutant^92^. Zebrafish are easily genetically manipulated, which has led to the generation of genetic lines that enable the study of ageing disease states in zebrafish^93,94^. Additionally, they are amenable to high-throughput drug screening in vivo, with larvae fitting in standard 96-well plates and reports of 500,000 zebrafish being screened in a single study^95,96^.

In the context of senescence, zebrafish have been reported to age in a telomere-dependent manner with shortening telomeres with age, a feature which is seen in humans but not widely observed in laboratory mice^97,98^. Additionally, zebrafish utilise telomerase reverse transcriptase (*tert)* to elongate telomeres unlike drosophila, which use a retrotransposon system^99,100^. Indeed, genetic alterations of *tert* have been carried out in zebrafish, for example, the hu3430 strain has a mutated *tert* gene that prevents its full-length translation^101^. Without full-length TERT expression, telomerase is inactive in these fish which causes a premature ageing phenotype^97^. This is useful for characterising the mechanistic effects of ageing as zebrafish, for example, those homozygous for the hu3430 alleles exhibit a more rapid build-up of senescent cells. Additionally, rescuing the expression of TERT in the intestine alone was sufficient to recover a significant amount of the premature ageing effects observed^102^. This indicates that zebrafish can be used as an effective model of age-related senescent cell accumulation and that their ageing phenotype can be altered using genetic manipulation. Other senescence markers that are described in zebrafish include lipofuscin accumulation, mitochondrial dysfunction, SA-β-Gal and the DNA damage response^103–108^. Physiological roles of senescence in embryonic development and wound healing are also evolutionarily conserved in zebrafish^109,110^.

Further, recent work has produced a new zebrafish reporter line that may be used for assessing senescence and testing the effectiveness of novel senotherapies in vivo^68^. Morsli et al. developed a p21:GFP reporter line which allows the live in vivo quantification of senescent cell burden in zebrafish larvae using fluorescent imaging techniques and fluorescence-associated cell sorting (FACS). Using γ-irradiation, premature senescence was assessed by increases in SA-β-Gal, p21, p53, p16-like (a P16 orthologue), γH2AX, IL-6 and reduced proliferation. The group observed a significant decrease in the number of senescent cells when the larvae were treated with the known senolytics Dasatinib and Quercetin (D + Q). This was determined through reduced GFP fluorescence, detected in the Opera Phenix™ high-content screening system. Due to the high fecundity of zebrafish and the rapid nature of this in vivo assay, this line may provide a rapid method for screening novel senolytic therapies in a vertebrate model organism^96^. An additional benefit to screening in transgenic zebrafish such as these, is that on- and off- toxicities will be apparent as they are being assessed in the whole organism context^96^. Additionally, behavioural tests of organism health such as swimming ability and cognitive function are feasible in zebrafish^111^. These types of screening are not feasible in vitro and are far more costly in time, labour and financial burden in mice. Overall, zebrafish are an effective model for studying senescence and therapies aimed at alleviating the senescent cell burden and can complement mammalian model systems.

Urodele amphibians, or salamanders, such as axolotls (*Ambystoma mexicanum*) and newts (*Notophthalmus viridescens*), possess an exceptional ability to regenerate complex body parts, including entire limbs, a process that begins with the formation of a blastema at the site of injury^112^. The blastema consists primarily of mesenchymal progenitor cells, which differentiate to develop and replace the structures and functions of the missing tissues^112^. Previous work has demonstrated that the blastema in both newts and axolotls contains a significant number of SA-b-gal-positive cells, which appear at distinct stages of regeneration^113,114^ and occur consistently with repeated injury^113^, demonstrating that senescence plays an essential role in the regenerative capability of these organisms. Interestingly, Yun et al. noted that the accumulation of senescent cells in newts appeared exclusive to the regeneration of lost tissues/structures, with no significant induction of senescence during normal limb development^113^, and observed no accumulation of senescent cells with age or repeated injury. These results are striking when compared to studied examples of senescence in mammals and other organisms, where the accumulation of senescent cells is considered a fundamental component of ageing^115^. Although it is unclear how the accumulation of senescent cells is prevented, salamanders appear to possess an efficient mechanism of senescent cell removal by macrophage-driven clearance^113^. Using an in vitro cultured axolotl cell line, further work demonstrated that the implantation of senescent cells into regenerating tissues enhanced the growth of the blastema through the action of paracrine factors, including members of the wingless-related integration site (Wnt) family, which may play essential roles in promoting the outgrowth of the blastema and thus generating a cellular niche which promotes the regenerative process^114^.

Additionally, in axolotl and African claw frog (*Xenopus laevis*) development, senescent cells were detected during the degeneration of the pronephros and the appearance of mesonephros^116,117^. This was detected via SA-β-gal staining and the absence of proliferation markers such as Ki67 and EdU incorporation. Senescent cells were also observed in several other anatomical areas, including the gums and lateral organs of axolotls and the mid/hindbrain and cement glands of African claw frogs^116,117^. Davaapil et al. further demonstrated that the induction of senescence in axolotl pronephros was delayed by inhibition of TGFβ, confirming that cellular senescence is a programmed step in embryogenesis and controlled through similar mechanisms as those observed in mammals^118^. When one considers that salamanders also exhibit a remarkably long lifespan compared to organisms of a similar size, an uncommonly low incidence of cancer^119^, and potentially similar mechanisms of senescence induction as mammals, the study of cellular senescence in salamanders, as well as other amphibians, is of particular importance to the field. Comparatively poorly studied, further investigation into these model organisms will lead to new insights and therapeutic approaches for other organisms with limited regenerative capacity, cancer resistance, and longevity, including mammals.

## Mammalian models of senescence

### Progeric mouse models

In ageing research, accelerated ageing models play a crucial role as widely employed experimental systems (Fig. 1). Progeroid syndrome is a rare disease with a wide range of pathological manifestations associated with premature ageing^120,121^. As a result, individuals with these diseases typically have a shortened lifespan and early onset of osteoporosis, cardiovascular alterations, and hair loss, among other disease-specific features^122^. These diseases are mainly caused by laminopathies and changes in the DNA repair machinery, which will be discussed below^123^. However, not all mutations in genome integrity maintenance pathways or DNA repair machinery lead to premature ageing.

These accelerated ageing models have been associated with an increased burden of senescent cells in human patients with progeroid syndromes and in mouse tissues^66,124–132^. In contrast to nematode worms, fruit flies and killifish, ageing studies in mice take years. The benefits of utilising progeroid mouse models include the rapid onset of the phenotype, the direct association of the manipulated gene and the alterations observed, as the majority of the models imply single gene deletions^120^. Additionally, genetically manipulated mice exhibit distinctive and reproducible features in contrast to naturally aged wild-type mice^120^. These models also have certain limitations. For instance, the results of artificially introduced pathogenic variants in physiological ageing should be carefully extrapolated^133^. Furthermore, there is a lack of correlation between the accelerated ageing pattern observed in particular organs and that observed in the human-aged counterpart in certain progeroid models^123^.

Additionally, although this is not specific to progeroid mice, the sex of progeroid mice in experiments is often underreported^123^. This represents a challenge in the interpretation of the results as differential DNA double-strand break repair in the course of ageing has been reported in both sexes^134,135^, a fundamental process in the preservation of genome stability. This issue about the understudied sex differences and the potential implications in senotherapy will be further discussed later.

In progeroid mouse models of DNA repair deficiency, the expression of BUBR1 results in the generation of a kinase involved in mitotic spindle assembly, which is a critical factor in the correct segregation of chromosomes. Aneuploidies and incorrect chromosomal segregation are the result of deficiencies in BUBR1 expression^66^. Knockout mice are lethal, therefore hypomorphic models that preserve 10% of the protein expression, designated *BUBR1*^*H/H*^, are employed as an alternative. The estimated lifespan is approximately six months^123^. Baker et al. demonstrated for the first time that the clearance of p16+ cells reduced age-related physiological deterioration in a *BUBR1*^*H/H*^ mouse line^13^. The ERCC1-XPF enzyme complex plays a role in the repair of DNA via the excision of nucleosides. The complete knockout of ERCC1 has been observed to result in a lifespan of 3–4 weeks, while the hypomorphic variant *ERCC1*^−^^*/Δ*^, which preserves 10% of protein expression, has been found to exhibit a lifespan of 7 months^66,136^. The *Polγ*^*D257A/D257A*^ model demonstrated a reduction in the proofreading activity of Polγ, which is involved in mitochondrial DNA replication. As a result, the mice displayed an accumulation of mitochondrial DNA. The lifespan of the subjects ranged from 48 to 61 weeks^137^. *Sod1*^−^^*/*−^ mice exhibit accelerated ageing in conjunction with oxidative stress, in accordance with the theory of ageing which posits a link between this process and the accumulation of ROS^138^. The model exhibited a 30% reduction in lifespan that can be extended if mitochondrial catalase is overexpressed^66^. XPD gene is associated with nucleotide excision DNA repair therefore *Xpd*^*TTD/TTD*^ mouse model posses accumulation of DNA damage and accelerated ageing^139^.

With regard to laminopathies, truncated variants of lamin A, designated progerin, accumulate as a consequence of mutations in the *LMNA* or *ZMPSTE24* genes^140,141^. The mutations may be either the loss of the ZMPSTE24 cleavage site or the loss of function of this protein, both of which result in the retention of highly stable, sub-processed farnesylated precursor prelamin A^142^. Among the various progeroid mouse models that are based on this mechanism, it is noteworthy to mention *Lmna*^HG/+^ whose lifespan is around 6–7 months. These mice share several significant phenotypic features with Hutchinson-Gilford progeria syndrome and gradually develop characteristics such as hair loss, impaired growth, bone density loss, and reduced subcutaneous adipose tissue, among others^140^. Full lamin A and C knockout (*Lmna*^−/−^) mice exhibit a lifespan of 6–7 weeks, while the *Lmna*^LCO^ mouse model, which synthesises lamin C but not lamin A, does not present any pathological phenotype. This suggests that a deficiency in lamin A can be compensated by the presence of lamin C. *Zmpste24*^−/−^ mice present a 20-week lifespan with distinctive osteolytic lesions that drive rib fractures^140,143,144^. It was recently generated a *Lmna*^L648R/L648R^ mouse model that possesses a substitution in ZMPSTE24 cleavage site in prelamin A that exhibits an increased lifespan in comparison to the null knockout, which can reach 2 years. This model can offer the advantage of studying the pathological accumulation of prelamin A within the framework of physiological ageing^142^.

There are also described non-progeroid mouse models of accelerated ageing. For example, klotho deficient mice possess premature ageing-like characteristics such as skin and muscle atrophy, soft tissue calcifications, kyphosis, atherosclerosis, osteoporosis, and pathological mineral ion metabolism, among others. The subjacent mechanism involved in accelerated ageing has been proposed as part of an altered regulation of fibroblast growth factor 23 (FGF23) signalling that has a central role in ion homoeostasis^145^. Additionally, the Senescence-accelerated mice P8 (SAMP8)is characterised by accelerated brain ageing features that resemble what is seen in humans, making it a suitable model for neurodegenerative disorders such as dementia and Alzheimer^146^.

### Slow ageing mammalian models

Slow-ageing mammals provide valuable natural models for the study of ageing, although practical challenges arise from the need for long-term monitoring across all life stages^147^. Bats are long-lived mammals, exceeding the expected lifespan for their body size and metabolic rate by at least threefold. Bat metabolism can be artificially modified by temperature, and they exhibit hibernation patterns. Their average lifespan ranges from 20 to 30 years, posing challenges for accurate age determination in adult individuals^148^. Notably, bats display negligible senescence and remarkable resilience to cellular damage^149^. Consistent with the oxidative damage theory of ageing, various studies have reported enhanced oxidative protection, DNA repair mechanisms, and tumour suppression in bats compared to other mammals^150^. Similar to humans, bats typically show decreased telomere length with age^151^.

Naked mole rats, the most long-lived rodents, can live for up to 37 years and represent another valuable natural model to study ageing. These animals exhibit features such as natural adaptations to hypoxic environments, a slow decline in fertility, and a poor inflammatory response against carcinogenesis inductors^152^. As in the case of bats, DNA repair pathways were found to be upregulated in comparison to those in mouse models^153,154^. Notably, increased basal levels of oxidative stress are found, even though they are not associated with an increase in antioxidant capacity, which would not explain the increase in longevity^155^. There are discrepancies among reports regarding the sensitivity of cultured cells derived from naked mole rats and the way they undergo senescence when triggered by different stimuli. It has been reported that a higher dosage of irradiation is required to induce senescence in naked mole rat fibroblasts in comparison to mouse counterparts^156^. Kawamura et al. describe a mechanism observed in vitro and in vivo in naked mole rats in which the accumulation of senescent cells is prevented by specific cell death induction, thus representing a natural senolysis phenotype. Naked mole rat fibroblasts concentrate serotonin under basal conditions and when senescence is induced, the INK4a-Rb pathway is upregulated, generating an increase in Monoamine Oxidase (MAO) enzymes that utilise serotonin to produce H_2_O_2_ to trigger cell death. This mechanism was not observed in mouse cells, indicating that the efficient natural elimination of senescent cells, together with the resistance to the induction of senescence as suggested by the authors in the early and late stages of the animal life cycle, could explain the long lifespan of this animal^157^. The study of long-lived mammalian species in senescence and ageing could provide novel mechanisms that limit human lifespan.

### Transgenic mouse models for p16

Considering the crucial role of senescence-associated markers in ageing pathology, various models have been developed by engineering the expression of p16 via transgenic approaches in mice. The first of these models came in 2011, where the researchers generated an INK-ATTAC (*Ink4a* apoptosis through targeted activation of caspase) transgenic reporter. Here, p16-expressing cells activate the expression of enhanced Green Fluorescence Protein (eGFP) together with an engineered fusion protein (FKBP-Casp8) that permits the specific detection and elimination of p16-expressing cells^13^. This was the first time that p16 accumulation was causally linked to ageing phenotypes, albeit in a progeric model. A follow-up study showed comparable results in a wild-type ageing mouse^158^. An alternative transgene using luciferase was engineered under the p16 promoter^159^. This reporter correlated luciferase signal with age and was able to detect spontaneous tumorigenesis in vivo. Further, the P16-3MR model was published in 2014^70^. This model contains a 3MR (modality reporter) fusion protein which consists of functional domains of a synthetic Renilla luciferase (LUC), monomeric red fluorescent protein (mRFP), and truncated herpes simplex virus 1 (HSV-1) thymidine kinase (HSV-TK). This model allowed the detection of p16-positive cells in vivo and the clearance of these cells via ganciclovir (GCV) administration and demonstrated an essential role for senescence in wound healing in mice. Recently, a preprint demonstrated unspecific luminescence from p16-3MR mice in multiple models of senescence, which should be considered going forward^160^. An additional model developed by Liu et al. assessed p16 promoter activation in mice via the addition of a tdTomato fluorophore into exon 1 of the p16 locus^161^. Here, the researchers found that tdTomato-positive cells increased with age, and when applied to a model of peritoneal inflammation, observed tdTomato+ macrophages with reduced proliferation, SA-β-Gal, and some SASP transcripts.

In 2020, Grosse et al. generated a knock-in mouse line integrating CRE recombinase, thymidine kinase (TK), and a fluorescent reporter (tdTomato) cassette at the end of exon 3 within *cdkn2a* gene^60^. The use of self-cleaving peptides in the CRE recombinase cassette allowed the production of separate proteins. This knock-in line was bred with *Rosa26-mTmG* and was such that all cells were positive for tdTomato until p16-driven CRE recombinase switched the red fluorescent signal to EGFP in p16-positive cells only^60^. The researchers demonstrated that the removal of p16-positive vascular endothelial cells deteriorated blood-tissue barriers and mouse health. In the same year, Omori et al. published the development of p16-Cre^ERT2^-tdTomato mice where a tamoxifen-inducible Cre recombinase system was introduced into the first exon of the endogenous p16 locus^162^. The generated p16^Ink4a^-Cre^ERT2^neo mice were then paired with the Rosa26-CAG-lsl-tdTomato line which allowed effective labelling of p16-high cells. The group also introduced a Diphtheria Toxin Receptor (DTR) element to specifically ablate p16-high cells, which resulted in reduced steatosis and liver inflammation in a nonalcoholic steatohepatitis (NASH) model.

INK4a H2B-GFP reporter-in-tandem (INKBRITE) was created as a highly sensitive fluorescent reporter for p16 promoter expression. This mouse model used a Bacterial Artificial Chromosome (BAC) with tandem cassettes of GFP fused in frame with the p16 locus, resulting in multiple copies of the fluorophore targeted to the nucleosome. They used this reporter to show that p16+-positive cells are required for the maintenance of a reparative niche in the lung and help to augment epithelial repair upon injury^58^.

The p16-FDR mouse model was established to understand the role of p16-positive cells in lung carcinogenesis^163^. In this model, a multifunctional transgene was knocked into the p16 locus such that tdTomato was expressed as a fusion to DTR, allowing dual identification and clearance of p16-positive cells. Upon activation of oncogenic KRas, the p16-FDR mouse showed increased tdTomato+ fluorescence during the early stages of tumorigenesis, particularly in macrophages and endothelial cells. Upon ablation of these cells, the pathology of these adenocarcinomas was reduced. This finding was also mirrored using the INK-ATTAC mouse model mentioned prior^164^.

### Transgenic mouse models for p21

Fundamental discoveries in senescence have been made with transgenic mouse models of p16. However, due to the heterogeneity of senescence mentioned previously, it is not sufficient for a holistic understanding of the phenotype. For example, p21 expression can lead to alternative and unique phenotypes that are not always associated with p16 expression^59,63^. As such, transgenic reporters for p21 have proved synergistic.

A mouse with a tamoxifen-inducible Cre-ERT2 recombinase knocked in at the p21 locus provided a flexible approach to researching the gene through breeding with other transgenic reporters^62^. For example, when bred to a loxP-STOP-loxP luciferase reporter mouse, both transgenes could monitor live p21 expression dynamics through the in vivo imaging system (IVIS). The p21-Cre mouse was crossed with a loxP-STOP-loxP tdTomato reporter mouse and allowed detection and isolation of tdTomato+, p21+, cells with age, obesity and chemotherapy treatment by immunofluorescence and flow cytometry. When instead crossed with a loxP-STOP-loxP Diphtheria Toxin A mouse, the p21-positive cells could be genetically ablated. This resulted in improved physical function in ageing and obesity models. Finally, as a proof of concept, the SASP of p21-positive cells could also be specifically ablated in the p21-cre mouse crossed a model allowing Cre-dependent deletion of the NF-κB subunit *RelA*^165^. An additional p21-Cre mouse was developed and demonstrated that p21^HIGH^ are distinct from P16^HIGH^ cells and that monthly clearance of p21^HIGH^ cells in mice from 20 months of age extended lifespan and function in multiple tissues such as the heart and liver^165^.

To directly compare the relative contributions to in vivo senescence, a p21-ATTAC mouse model was created to complement the p16 INK-ATTAC mouse model^13,59^. p21 promoter activation is detected by eGFP and p21-positive cells can be ablated through caspase 8 driven apoptosis by AP20187. Here, a model of osteoporosis using 24 Gy of focal radiation treatment (FRT) in a 5 mm area of the right femoral metaphysis was used to compare both transgenic models. In both ATTAC mice, AP20187 removed eGFP-positive cells following radiation, however only in the p21-ATTAC mouse was there prevention of radiation-induced osteoporosis. Additionally, the levels of inflammatory SASP factors were only reduced following p21+ cell ablation.

In a similar vein, a p21-3MR mouse model was created that complements the already established p16-3MR mouse^70,166^. With the 3MR transgene in the endogenous p21 promoter region, p21+ cells were able to be genetically ablated with Ganciclovir (GCV) treatment. This prevented doxorubicin-induced weight loss in vivo suggesting a role for p21+ cells in adverse effects of chemotherapy treatment. The RFP component of the 3MR transgene showed increased fluorescence following doxorubicin treatment, which was removed with GCV co-treatment and correlated with improvements in tissue histology. These findings demonstrate the need for assessment of senescence using multiple approaches to improve our overall understanding of the phenotype in vivo.

### Transgenic mouse model for p19

To assess the role of senescence in lung pathology, p19ARF-DTR mice were developed^167,168^. p19ARF - p53 signalling pathway, though less explored than p21 and p16, is also a central tumour suppressor mechanism involved in senescence. p19ARF-DTR mice carry an extra CDKN2A allele altered to express firefly luciferase and the human diphtheria toxin receptor under p19ARF regulation. This allowed live detection of senescent cells and ablation of p19ARF cells improved pulmonary function and protected against pulmonary emphysema. A protocol for the assessment of lung senescence in this model has been published^169^.

### Transgenic mouse model for Glb1

In an alternative approach, a Glb1-2A-mCherry (GAC) reporter allele, produced in 2022, was developed to act as an in vivo lysosomal β-galactosidase reporter^170^. In this report, the authors have used the increased expression of *Glb1*, the gene encoding β-galactosidase, as an indicator of altered senescence in vivo. The self-cleaving 2A peptide allowed the labelling of highly expressing *glb1* cells with mCherry in vivo. Here the authors show that the *Glb1* mCherry signal can predict lifespan in middle age, though this correlation was not seen in later life. Exposure to bleomycin, known to induce lung fibrosis and senescence, was able to increase the mCherry signal seen in the lung, whilst senolytic DQ therapy reduced the mCherry signal and other markers of senescence and fibrotic histopathology. A limitation of the use of this genetic reporter is that it is known that *Glb1* inhibition, unlike p21 or p16, does not alter the senescence phenotype and so its direct role in senescent cells is questionable^171^. Nevertheless, this is a welcome addition to the wealth of in vivo tools available to study senescence.

### Transgenic mouse model for GPNMB

Suda and colleagues successfully revealed Glycoprotein nonmetastatic melanoma protein B (GPNMB), which was initially found in melanoma cell lines, as a transmembrane protein that is highly expressed in senescent endothelial cells^172^. Senescent endothelial cells, similar to other ageing cells, accumulate in tissues during ageing and in conditions such as atherosclerosis^173,174^. These senescent cells exhibit abnormalities, such as insulin resistance, driven by the SASP^175^. The authors generated a Gpnmb-DTR-luciferase transgenic mouse model that Gpnmb-positive cells can be tracked with luciferase signals, while diphtheria toxin has been used to eliminate these cells^172^. Thus targeting vascular ageing might provide health benefits. When subjected to mice with a high-fat diet, luciferase signals were abundant in visceral fat; Conversely, this toxin treatment diminished this activity. Additionally, the elimination of Gpnmb-positive cells also resulted in a reduction of atherosclerotic plaque formation. These findings indicate Gpnmb as a potential target for senolytic therapy.

### Rat models of senescence

While mice take centre stage when it comes to mammalian models of senescence, a niche has been carved out for rats with some interesting models produced. For example, the senescence-accelerated OXYS rats are a strain of Wistar rats selectively bred so that they spontaneously develop accelerated senescence making them useful for the study of senescence and associated diseases^176^. Alternatively, the Dahl salt-sensitive Obese (DS/Obese) line has been established that contains a mutation in the leptin receptor gene (*Lepr)* that leads to an onset of metabolic syndrome when the rats are fed on a normal diet^177^. DS/Obese rats were found to have increased fibrosis, inflammation, and oxidative stress compared to lean controls. Takahashi et al. found that these negative effects led to a premature onset of myocardial senescence, indicated by upregulation of senescence-associated genes including *p21, p53*, and *Chk2*. Further, aged rats (>22 months old) have been found to be suitable for assessing the effectiveness of senotherapeutics. For example, Krzystyniak et al. found that aged rats treated with the dual regime of Dasatinib and Quercetin for 8 weeks alleviated deficits in memory impairment observed in aged rats^178^.

## Future perspectives of novel senescence characteristics

There is a pressing need in the field for models that utilise multiple senescence markers to enhance the characterisation of senescent cells in living organisms. The current gold standard is to use a multitude of senescence markers and as such there are limitations to the use of transgenic models that rely on a single marker^19,20^. Though, it would prove far more difficult and costly to create, breed, maintain and validate a mouse model with multiple transgenic alleles for senescence identification.

A further consideration is that senescent cell autofluorescence can contribute to difficulties in their identification using fluorescent reporters in vivo. Fortunately, the development of spectral cell analysers in recent years allows for better identification of true fluorescent signals by flow cytometry^179^. Moreover, fluorescent proteins such as GFP can themselves have toxicity and immunogenicity that could confound the results from such transgenic animals^180^. The Cre-loxP system can be leaky also, with endogenous loxP sites in the genome potentially also leading to confounding variables^181^. Therefore, unbiased assessment without using fluorescent reporters for individual proteins could better distinguish the true in vivo heterogeneity of senescent cells. The emergence of new technologies, particularly with single-cell resolution, gives the opportunity for a deeper analysis for better identification of senescent subtypes. For example, as senescent cells are often implicated in a DNA damaged or tumorigenic context with multiple genetic alterations, deeper and longer-read single-cell sequencing methods such as SmartSeq3 should provide a greater contextual understanding of splice variants in senescent subtypes^182^. Additionally, expanding outside of transcriptomics will provide crucial evidence for senescent cell biology. Single-cell nucleosome-methylation and transcription sequencing (sc-NMTseq) is one such published method that provides single-cell multiomics^183^. Spatial omics technology has progressed to nearer single-cell resolution and can provide an opportunity to see the involvement of senescence in the tissue architecture, and how cells organise around them^184^. In all, the characterisation of senescent cells is becoming more nuanced over time with improved in vivo models and technologies. This will inevitably allow better clinical translation for the improved diagnostic and therapeutic potential of senescence research.

## Diagnostic identification of senescence and clinical implications

A vital avenue for the translation of senescence research to the clinic is the development of tools to identify senescence burden diagnostically. Traditional approaches involve biopsy samples and can only provide a snapshot of the senescence burden in a particular tissue at a particular time. Additionally, a tissue biopsy will only provide accurate information about the local area and not the overall burden of an organ or heterogeneous disease^185^. Several protocols have been published to detect senescence in vivo for more reliable assessment of clinical specimens^19,20^, and a recent guideline was developed to identify the distinctive features of senescent cells in living organisms and tissues^186^. Altogether these efforts help to standardise our approaches, though, as robust analyses of the contextual nuances of senescence show their diversity, our understanding remains somewhat generic. To advance, we must have clear and ideally distinct single or combinations of markers for senescence that would identify whether it is skewed to a detrimental or beneficial response clinically. Significant resources are being placed in this area, such as in the creation of The National Institutes of Health (NIH) Cellular Senescence Network (SenNet) Consortium (SenNetT)^187^. SenNet aims to map senescent cells using single-cell technologies across mouse and human lifespans in multiple tissues. Recently, they published an expert recommendation for the detection of senescence in different cell and tissue types^188^. It would prove extremely beneficial to appreciate the burden of senescence through clinically accessible means and in a context-specific manner. It is extensively described that senescent cells are causally linked to many diseases pre-clinically, and there are many clinical trials being carried out to translate this into human pathology^189^. To complement this, a great deal of effort has gone into developing diagnostic probes to identify senescent cells using probes and in biological fluids. We will explore these below, expanding on their advantages, limitations, and amenability to clinical translation.

## Senoprobes

### SA-β-Gal activity

Diagnostic probes to detect senescence (‘Senoprobes’) with considerable preclinical specificity have been created^190–192^. The predominant subtype currently uses the increased lysosomal SA-β-Gal activity in senescence to induce a detectable and sometimes clinically relevant signal.

The initial approach for monitoring SA-β-Gal activity involved using a synthetic β-Gal substrate called X-Gal (5-bromo-4-chloro-3-indolyl-β-d-galactopyranoside) in fresh fixed cells and tissues. This colorimetric assay provided a population-level snapshot in vitro and in vivo, with aforementioned limitations^193^. Recently, reflected light microscopy of X-Gal crystals in adipocytes allowed superior detection and quantification over traditional brightfield imaging, and it is compatible with immunofluorescence^194^. To achieve improved resolution, a fluorescent substrate for β-Gal, called 5-dodecanoylaminofluorescein di-β-D-galactopyranoside (C12FDG), was developed^195^. This cell-permeable substrate emitted green fluorescence and remained intracellular upon cleavage by β-Galactosidase. However, drawbacks of this approach included low tissue penetrance, in vivo autofluorescence, low cell loading, and a slow response rate. A far-red shifted version of the fluorescent substrate, called 9H-(1,3-dichloro-9,9-dimethylacridin-2-one-7-yl) β-d-galactopyranoside (DDAOG), showed improved autofluorescence and tissue penetrance^196^. Despite these improvements, more work was needed for effective in vivo assessment in most tissues^194^. SPiDER-β-Gal was developed as a fluorescent substrate allowing live single-cell resolution detection of SA-β-Gal activity, with improved cell permeability compared to C12FDG^197^. Once activated, the reactive quinone methide on SPiDER-β-Gal caused binding of the fluorescent substrate to intracellular proteins, resulting in improved intracellular retention of the fluorescent signal compared to C12FDG after washing or fixing cells.

To enhance tissue penetrance and reduce photodamage of SA-β-Gal-based senoprobes, a two-photon approach was developed using near-infra-red light for quantitative assessment of enzymatic activity^198^. The SG1 probe responded to β-Galactosidase activity faster than C12FDG and was more sensitive. However, even with an improved tissue depth of 140μm, in vivo translation remained challenging. Detection of SA-β-Gal activity in live cells was achieved using a pro-version of a β-Galactosidase substrate called Gal-Pro^199^. This probe did not emit any near-infra-red (NIR) fluorescence until its glycosidic bond was cleaved by β-Galactosidase, resulting in a rapid fluorescence turn-on at a 703 nm emission wavelength. The signal-to-noise ratio was improved compared to C12FDG, enabling the detection of senescence in live cells, but further in vivo translation is warranted. An OFF-ON two-photon senoprobe was developed to further improve tissue penetrance and in vivo translatability^200^. AHGa encompasses a naphthalimide fluorophore that emits strong fluorescence at 540 nm upon cleavage by β-Galactosidase. Fluorescence was detectable in palbociclib-treated SK-MEL-103 murine xenograft tumours compared to vehicle-treated controls after sacrifice. Further work to detect live in vivo senescence was required. The two-photon approach was further developed with HeckGal^201^. Once cleaved into the fluorophore Heck in the presence of SA-β-Gal activity, it is detectable in ex vivo models of kidney fibrosis from folic acid treatment and in an orthotopic breast cancer model treated with palbociclib.

Live in vivo assessment of SA-β-Gal activity was recently achieved with a novel probe called QM-β-Gal alongside optical fibre confocal imaging^202,203^. QM-β-Gal is an Aggregation-Induced Emission luminogen (AIEgen) that, upon β-Gal activity, creates fluorescent nanoaggregates. Doxorubicin-treated breast cancer transplanted (MDA-MB-231) mice injected with QM-β-Gal showed a rapid increase in fluorescence within the first hour following probe injection and persisted for at least 24 h, the latest time point assessed. Importantly, fluorescence from QM-β-Gal aggregates was reduced by ABT-263 (Navitoclax) therapy from 3 days post-treatment, with continuous treatment further reducing fluorescence over 14 days. This reduction correlated with decreased tumour size compared to doxorubicin treatment alone, demonstrating that with more sophisticated imaging techniques, live changes in senescence burden can be monitored in vivo using QM-β-Gal.

A recent study developed a fluorogenic sulfonic-Cy7Gal probe for the detection of SA-β-Gal activity in vivo using more standard imaging methods^204^. In the presence of β-Galactosidase activity, the non-emissive probe is hydrolysed to release a highly fluorescent Sulfonic-Cy7 dye that diffuses out of the cell and is cleared by the kidneys. As a result, fluorescent signals are detectable and quantifiable in the urine. In female mice bearing 4T1 tumours, Sulfonic-Cy7 fluorescence was detected live by IVIS in the bladder following palbociclib treatment. The fluorescent signal was also detected slightly in the tumour ex vivo, though at a much lower intensity than in the bladder. With age, an increased fluorescence signal could be seen in 14-month-old mice compared to 2-month-old controls. When comparing aged matched senescence-accelerated and senescence-resistant mice, senescence-prone mice showed increased Sulfonic-Cy7 fluorescence that correlated with increasing p16 and decreasing lamin-b in the kidney & liver. Finally, 15-month-old mice were treated with the senolytic combination of D&Q, Sulfonic-Cy7 fluorescence was reduced in the treated mice. After 58 days following a senolytic regimen, the fluorescent signal returned to control levels, indicating that intermittent senolytic therapy may not provide long-term reduction in senescence. This demonstrates a novel non-invasive approach for monitoring in vivo SA-β-Gal activity, enabling longitudinal studies. One consideration is that as the signal is in the urine, the source of the signal must be confirmed.

An alternative approach for detecting SA-β-Gal activity using Mesoporous silica nanoparticles (MSNs) has been established^205^. An MSN coated with a galacto-oligosaccharide capable of being cleaved by β-Galactosidase activity, termed GosNP, was a basis for many early nanoparticle-based senoprobes. This approach was further developed by instead using a homogeneous 6‐mer galacto‐oligosaccharide coated MSNs, termed GalNP^206^. Rhodamine-loaded GalNP selectively identified cells with increased SA-β-Gal activity in vitro, and ex vivo both in tumours from Palbociclib-treated mice and bleomycin-treated fibrotic lungs. A dye approved by the FDA for human use, with advantageous in vivo imaging properties, was instead used as cargo for these MSNs^207^. Nile Blue loaded β-Gal activatable MSNs were detectable in vivo by IVIS imaging in Palbociclib-treated mice, with practically no signal in other organs ex vivo^208^. Alternative nanosystems for combined detection and elimination of SA-β-Gal-positive cells have been developed^209^. Gal-(ZnPc*)_2_-NP self-assembles in aqueous media, but in the presence of β-Gal activity will disassemble and release photoactive monomeric phthalocyanine units that can be detected in HeLa cells by its fluorescent signal, and simultaneously kill the cells through increasing intracellular Reactive Oxygen Species (ROS). This has yet to be translated in vivo.

Fluorescent probes to detect senescence markers show promise in the preclinical setting, however their uses are limited in the clinic due to fluorescent signal penetration depth, or the requirement of specialised imaging techniques for live in vivo imaging^203^. As a result, more clinically relevant imaging approaches to assess β-Gal activity have already been established with Positive Emission Tomography (PET), Magnetic Resonance Imaging (MRI) and Nuclear Magnetic Resonance (NMR) imaging^210–212^. Adjustments can feasibly be made to these MRI and NMR based probes to improve specificity to senescent cells^213^. The most clinically relevant approach for tracking live SA-β-Gal activity non-invasively in vivo is through the use of a PET tracer [18F]FPyGal^214–216^. This type of imaging is advantageous over fluorescence probes in that it can be used to identify changes much deeper in the tissue, in a non-invasive manner^217^. However, this type of imaging is at the population level and conceivably not at cellular resolution at this time, and so is potentially useful in a context where there is a large change in senescence burden. This is patented (US20190381198) and clinical trials are under way to assess safety and efficacy (NCT04536454)^214,218^. Initially the safety and tolerability was assessed in a cohort of healthy volunteers. In the indicators assessed, the radiotracer was well tolerated and safe in the described population. The recent conference abstract described that in patients with rectum cancer, the [18F]FPyGal radiotracer correlated with histological detection of p21^Cip1^, p53, p16 and SA-β-Gal in surgically resected samples post-therapy, compared to treatment-naive biopsy samples. The proposed study also involves non-small cell lung cancer and adenocarcinoma of the esophagogastric junction, with a projected completion date of June 2024, we eagerly await these results. [18F]-PyGal has also been developed concurrently^213,219^. In 5-, 12- or 23-month-old mice, doxorubicin was injected into the knee joint, resulting in increased detection of [18F]-PyGal compared to controls that were also associated with increases in p16, p21 and SA-β-Gal. Senescent mesenchymal stem cells (MSCs) were injected into the knee cartilage of pigs, which also showed an increased [18F]-PyGal signal. Recently, an additional stable PET agent was established for the detection of SA-β-Gal in vivo, [68Ga]Ga-BGal^212^. Specific detection was observed in live mice with *LacZ* overexpressing CT26 tumours and doxorubicin-treated HeLa tumour xenografts.

### Alternative lysosomal reporters

It is well understood that detection of senescence by relying on lysosomal activity of SA-β-Gal alone is limiting sensitivity and specificity^26–28,171,220–222^. Senoprobes with potential for clinical translation are also being explored using alternative senescence markers.

Sialidase, which cleaves sialic acid (Sia) from glycoproteins and glycolipids and has functional roles in multiple tissue types, as well as being altered with age^223^. As the amount of Sia reduces with age, a potential increase in Sialidase activity could explain this. To test this, a probe was developed to identify whether Sialidase activity is a novel senescence marker^224^. Sia-RQ is a fluorescence quenched probe that, upon cleavage by Sialidase, will activate the ROX fluorophore for detection for in situ fluorescence labelling. Palbociclib-treated human hepatocarcinoma cells, Huh-7, showed an increase in fluorescence with Sia-RQ compared to untreated control, potentially associating Sialidase with therapy-induced senescence. Assessment of Sialidase in other contexts of senescence, how they relate to the phenotype, and in vivo characterisation are required.

α-L-fucosidase (α-fuc) has also been reported as an alternative lysosomal biomarker of senescence^225^. It is a lysosomal hydrolase with increased activity in senescent cells, including those with low SA-β-Gal activity. Knockdown of FUCA1 inhibited the senescence phenotype following treatment with an aurora-kinase B inhibitor, whereas inhibiting the β-Gal gene, GLB1, is known to not alter the senescence phenotype^171^. Its enzymatic activity allowed it to be functionalized into a novel senoprobe, QM-NHαfuc^226^. This is an Aggregation-Induced Emission luminogen (AIEgen), akin to QM-β-Gal^203^. Rather than relying on SA-β-Gal, QM-NHαfuc responds to α-fuc enzymatic activity, transforming the non-emitting sensor to release a sharp increase in 586 nm fluorescence emission. This senoprobe was able to detect senescence in vivo following an aurora-kinase B inhibitor in a xenograft model using the Maestro 2 imaging system. Increased fluorescence corresponded with well-described markers of senescence such as p21, p53 and reduced proliferation (Ki67).

An additional approach for live in vivo detection of senescence, with potential for clinical translation, is a novel nanoprobe called ‘NanoJagg’^227^. This probe is created by self-assembly of indocyanine green (ICG) dimers, which are an FDA approved contrast agent with fluorescent and Photoacoustic Tomography (PAT) properties. Nanojaggs preferentially accumulate in senescent lysosomes through endocytosis, though the exact mechanism for this preference is not currently understood. Nanojagg fluorescence is seen in multiple models of senescence in vitro and ex vivo such as Palbociclib-treated SK-Mel-103 mouse xenografts. A unique feature of this senoprobe is its PAT properties, which allow for live detection of Nanojagg in mice. PAT avoids the tissue penetrance limitations of fluorescence-based senoprobes and provides a greater clinical translatability as a result, with potential for longitudinal monitoring of local senescence burden.

### Cell surface markers

Antibody-drug conjugates against senescent-specific cell surface markers can deliver fluorescent probes to specifically identify senescent cells^228^. Beta-2-microglobulin (B2M) is an MHC class 1 protein that was also identified as a cell surface protein on senescent EJ cells, a bladder cancer cell line following both p21 or p16 overexpression^229^. Molecularly imprinted nanoparticles (nanoMIPs) against B2M were developed and showed increased fluorescent particle accumulation on p16-induced senescent EJ cells^230^. Mice treated with nanoMIPs intraperitoneally, intravenously, and via oral gavage did not show altered weight compared to pretreatment and control. Similarly, Blood Urea Nitrogen (BUN), Alanine aminotransferase (ALT), and Aspartate aminotransferase (AST) levels were stable in serum samples 14 days after treatment, suggesting that nanoMIPs are well tolerated in vivo. To permit live in vivo imaging of nanoMIPs, they were loaded with DyLightTM 800 NHS Ester and fluorescence was assessed by IVIS in 2mo vs. 11mo mice. A specific increase in fluorescence was seen in the older animals, localised predominantly to the jejunum of the intestine. To further characterise this fluorescence, senescence assessment of the fluorescent jejunum cells would be useful. This cell surface marker has also been used to develop senolytic approaches and will be discussed later^231–233^. It is worth noting, B2M is also associated with cancers regardless of senescence^234^. The breadth of its use as a senescence marker in different in vivo contexts must be further validated and explored before clinical translation.

CD9 is a tetraspanin involved in immunity and hematopoiesis, as well as having clinical implications in cancer and atherosclerosis^235–237^. It has also been associated as a senescent cell surface marker in endothelial cells^237–241^. While the majority of reports using this cell surface marker have focused on clearance of senescent cells, CD9-coated MSNs loaded with a Cy5.5 fluorophore showed potential diagnostic implications^239^. Apolipoprotein E -knockout (ApoE-KO) mice fed a high-fat diet to induce an atherosclerotic phenotype, showed Cy5.5 fluorescence specifically in fatty aortas compared to IGg loaded CD9 nanoparticles and other tissues ex vivo. An alternative approach to identifying senescent endothelial cells in atherosclerosis has been explored by utilising antibodies against VCAM1 to specify a nanocarrier loaded with the lipophilic red or near-infra-red (NIR) fluorescent dyes NR668 or Cy5.5-TPB^242^. Primary endothelial cells from porcine coronary arteries induced to senescence ex vivo by the pro-oxidant stimuli angiotensin II showed a co-localisation of the fluorescent dyes compared to untreated controls. Co-localisation of the dye and senescence reporters at the cellular level are required to confirm specificity of the nanocarrier, whilst in vivo assessment would further demonstrate translatability.

### Alternative intracellular markers

Iron homoeostasis is altered with age^243,244^. Recently, iron accumulation has been identified in senescent cells due to ferroptosis inhibition and iron-mediated autophagy^245–247^. Further, iron has been shown to drive the senescent phenotype and fibrosis in vivo^247,248^. Iron deposition is present in both kidney and lung fibrosis in mouse and human tissues. In this paper, iron exposure was sufficient to drive the senescent and SASP phenotype, and detecting iron via MRI could act as a biomarker of kidney fibrosis in mice. Suggesting that the detection of iron is a non-invasive way to measure fibrotic disease and possibly providing an indication of senescence burden, albeit indirectly. Indeed, microscale-Magnetic Resonance Relaxometry (µMRR) was able to detect live senescent mesenchymal stromal cells (MSCs) due to their altered iron metabolism in vitro^249^. It is vital to distinguish senescent from non-senescent MSCs clinically due to the reduced functional ability of MSCs from donors containing senescent MSCs, limiting their regenerative abilities. µMRR can detect intracellular levels of iron, such that the time for T2 relaxation is shorter where there is increased iron accumulation, this is what was seen in human mesenchymal stromal cells (MSCs) treated with Doxorubicin or TGF-β1. This correlated with increased SA-β-Gal and γH2AX, as well as reduced Edu staining. Further, the altered iron metabolism in senescent cells has been functionalized using Fe3O4 nanoparticles to deliver Quercetin to senescent cells in vitro^250^. The use of iron as a marker of senescence should be explored further for diagnostic identification of live senescence burden in a clinical setting.

The accumulation of lipofuscin has been recognised as a marker of ageing and senescence^251,252^. Lipofuscin is a pigmented aggregate from oxidised ‘cellular waste’ such as proteins, lipids and metals^253^. Lipofuscin is normally diluted through proliferation and as such it accumulates in post-mitotic or non-proliferative cells. Sudan Black B (SBB) binds to lipofuscin and has been used for the detection of lipids, but more recently was suggested as a method for detecting senescence^251^. A lipophilic, biotin-linked Sudan Black B (SBB) analogue called SenTraGor™ (GL13) was created to classify senescence burden in paraffin-embedded human samples, whereas classical SA-β-Gal staining requires freshly fixed tissue^254–256^. This was designed to give a greater signal-to-noise ratio through allowing antibody-enhanced detection.

In addition to the detection of lipofuscin in archival material, lipofuscin aggregates are highly autofluorescent that can allow for label-free flow cytometry detection of potentially senescent cells in vitro^257,258^. More recently, a two-step process for fluorescent detection of lipofuscin was also established^259^. This allowed detection of therapy-induced senescence in vitro where live senescent cells showed a 10-fold increase in green fluorescence compared to proliferating controls. Further exploration in vivo is required for translation. An additional fluorophore-conjugated probe based on SBB was developed for live detection of lipofuscin-positive senescent cells^260,261^. GLF16 is a hydrophilic fluorescent SBB analogue that, unlike GL13, can identify lipofuscin-positive senescent cells in one step without antibody conjugation and fixation. GLF16 fluorescence was detectable in multiple senescent cell types, co-localised with common senescence markers such as p21, p16, absence of Ki67, and was lost in the presence of the senolytic cocktail D&Q. Cells sorted according to GLF16 fluorescence demonstrated a senescence transcriptomic signature, further validating the probe for live isolation of senescent cells. Due to the lipophilic properties of GLF16 required for lipofuscin detection, the probe was not amenable to live cell detection alone. To address this, GLF16 was encapsulated into a copolymer micelle, termed m-GLF16. m-GLF16 fluorescence was increased in live hydrogen peroxide-treated human lung organoids. m-GLF16 fluorescence correlated with increased p21^Cip1^ and decreased Ki67 expression. Finally, fluorescence was detected in live animals following intravenous injection, in both a palbociclib-treated melanoma mouse model and bleomycin-induced lung fibrosis mouse model.

### Mitochondrial dysfunction

Mitochondrial dysfunction is well described in senescence and ageing^262,263^. Monoamine oxidases are mitochondrial enzymes (MAO-A & MAO-B) that oxidise bioamines and can release reactive oxygen species (ROS) as a result. MAO overactivation has been reported as a driver of senescence and is associated with age-associated pathologies, particularly in the heart and brain^264,265^. Two near-infra-red (NIR) probes to detect MAO-B activity as a marker of oxidative stress have been established^266^. MitoCy-NH2 and MitoHCy-NH2 are ratiometric probes located to the mitochondria through a triphenylphosphonium cation that contains a Heptamethine cyanine fluorophore that is released upon MAO-B activity. Thirty minutes following intracranial injection of MitoHCy-NH2 in BALB/c mice of different ages (1-, 6-, 12- and 24-months old) there was an age-dependent increase in probe detection in live animals via IVIS imaging. ex vivo assessment of the brains also correlated with MAO-B protein expression. Modulators of MAO, used in clinical practice for Parkinson’s Disease, were able to reduce the fluorescent signal from MitoCy-NH2 in aged mouse brains. The models used are known to be associated with senescence, though this was not directly tested here. One could postulate the use of this probe in combination with others to validate it directly to senescence in vivo.

### Combination probes

To further improve the accuracy of senoprobes to mark senescent cells, novel approaches have been developed that require multiple markers of senescence for senoprobe activity^267,268^. A tandem probe, PGal-FA, that requires both SA-β-Gal activity and formaldehyde (FA) expression in cells has been described^267^. Formaldehyde is a stress-related toxic metabolite that is associated with age-related diseases. PGal-FA is made of a hydrazonate group that nucleophilically reacts with FA triggering a fluorogenic response. This hydrazonate group is shielded by a βgal-sensitive galactosyl residue linked to it, such that it requires dual marker expression for fluorescence emission. OVCAR-3 cells that naturally overexpress β-Gal showed less PGal-FA fluorescence compared to bleomycin-treated A549s, while colorimetric X-Gal was unable to discriminate between the two. This highlights an improved specificity to SA-β-Gal. Additionally, frozen sections of lungs from bleomycin-instilled mice demonstrated an increased PGal-FA fluorescence compared to saline-instilled lungs. For further translation of this probe, both a deeper assessment of the role of FA in senescent cells and the reliability of it as a marker of senescence generally is needed, as well as further in vivo characterisation. In any case, the approach of using probes that require multiple markers to provide increased specificity to senescent cells must be explored further.

An additional dual-parameter recognition senoprobe, requiring two aforementioned senescence enzyme activities, SA-β-Gal and monoamine oxidase A (MAO-A), for fluorescence induction has been recently reported^268^. Pβgal showed increased fluorescence in multiple senescence induction methods in different cell types. Importantly, the probe was able to distinguish non-senescent expression of MAO-A and β-Gal in cancer cells overexpressing these enzymes, which was not possible in single-marker probes. Evidence for in vivo translation is needed, and likely the probe will need to be contextualised into age-associated disease models that are well associated with MAO-A activity. With both reporters, relying on fluorescence signals narrows the clinical translation due to the limited penetration depth of this approach^269^. For the future development of senoprobes, context-specific methods must be established. Functionalising them to multiple markers to detect pathological senescence may be a pragmatic approach for this once in vivo evidence is generated.

## Detection of senescence in biological fluids

For effective clinical translation of identification of senescence burden, methods to detect senescence using clinically accessible methods such as through liquid biopsies are essential^270^. The benefits are that it is more permissive to multi-sampling over time, to potentially get a temporal understanding of senescence dynamics. This could be vital for understanding disease recurrence or therapy resistance.

### Soluble SASP factors

Clinically, liquid biopsies are becoming a foundational diagnostic tool^270,271^. As components of the SASP are the typical drivers of paracrine senescent signalling, monitoring SASP proteins in the circulation have been proposed for potential clinical diagnostic use^11,20,31^. Many soluble SASP factors were previously identified as human plasma ageing markers and biomarkers of age-related disease^31^. Additionally, the detection of circulating SASP factors in patients predicted age, frailty, and adverse health outcomes following surgery^272^. Soluble senescent factors also predicted the risk of onset for major mobility disability in sedentary humans aged 70–89^273^. In patients over 65 with no, or one, chronic condition at baseline, the risk of death after 6.3 years correlated with soluble SASP factors such as GDF15, VEGF, MMP2, PARC, and RAGE^274^. These highlight the potential for a SASP liquid biopsy with clinical relevance.

Patients with idiopathic pulmonary fibrosis (IPF) were assessed for circulating senescence biomarkers compared to those without diagnosed lung pathology^275^. LASSO regression analysis, a machine learning approach, demonstrated that these factors accurately discern whether a patient was diagnosed with IPF or not. Further, circulating concentrations of these markers predicted lung function, measured by clinical and molecular outcome measures. Over a 6-year follow-up, six SASP-associated factors correlated with a higher risk of death, including activin A, IL8, GDF15, MMP7, MDC, and TARC.

It is important to note that the factors showing significant differences vary between studies, probably due to the heterogeneity of senescence and the SASP in differing pathologies, patient inclusion criteria, and outcomes assessed^31,63^. The causal link of circulating SASP factors to senescence burden and pathology at the local disease site must be assessed to directly link the changes in peripheral SASP factors to changes in pathological senescence. Overall, there is myriad correlative evidence of soluble factors associated with the SASP being used to predict pathological or morbidity outcomes in humans, warranting further exploration for potential translation into the clinic. A consensus on which markers and combinations thereof are associated with detrimental senescence in a particular pathology is needed.

### Alternative soluble senescence markers

Detection of lipofuscin in fluids as a diagnostic reporter of senescence has also been reported^256^. Compared to young individuals (21–25 years old), soluble lipofuscin, detected by chemiluminescence from GL13 (SenTraGor™), increased in the serum of healthy aged (65–94 years old) and those with age-associated pathologies such as cancer, dementia, heart failure, and rheumatoid arthritis. Additionally, soluble lipofuscin was increased in the follicular fluid of infertile women (33–46 years old) compared to younger donors (23–27 years old).

Other bodily fluids could also be used to detect senescence burden. It has been shown that senescent cells can reduce the amount of the geroprotective protein ɑ-klotho in mice^276^. This change is detectable both in the brain and urine after senescent cell transplantation. Senescent ablation through genetic or D&Q treatment restored ɑ-klotho in the kidney, brain, and urine. This was replicated in the urine of IPF patients who had 9 doses of D&Q over 3 weeks, where their ɑ-klotho increased. SASP factors were also assessed in the urine, showing a negative association with ɑ-klotho levels.

### Senescent extracellular vesicles and their cargo

Key mediators of the SASP outside of soluble factors are extracellular vesicles (EVs)^277–280^. EVs are enclosed, lipid bilayer particles secreted by cells acting as messengers by transporting material between cells and tissues. EV cargo is extremely heterogeneous and contextually dependent, including proteins, lipids, miRNAs, nucleic acids, telomeric, and mitochondrial DNA^279^. Many studies focused on EVs from the blood of aged versus young individuals, but this hasn’t been directly traced back to senescent cells^281^. Interestingly, EVs from the urine of patients showed an enrichment of common SASP factors such as IL8, GRO, and MCP-1 in those over 60 compared to those younger than 30^282^. Senescent cells upregulate EV release to signal to, and alter the phenotype of, neighbouring cells^283–287^. This includes inducing paracrine senescence. Senescent EVs are locally involved in cancer pathology, vascular calcification in vitro, and premature ageing of coronary artery endothelial cells ex vivo^277,288–291^. Though its exploration has been limited in the field of senescence, EVs are detectable in practically all bodily fluids, providing potential for their use in senescence diagnostics via liquid biopsy^292^.

An established cargo of EVs is miRNAs, which could also be assessed in liquid biopsies for senescence^279,293^. miRNAs are short non-coding RNAs that can influence cell phenotype and gene expression by binding to complementary RNAs^294,295^. miRNAs can modulate the expression of key senescence genes, and changes in their biogenesis can activate or inhibit the senescence programme and the SASP^296–303^. miRNAs can be produced by senescent cells themselves and are involved in the paracrine signalling that regulates phenotype and pathology^304,305^. A preprint from Weigl et al., demonstrates an in-depth profiling of miRNAs in response to doxorubicin treatment in vitro in multiple cell types, and senescence-inducing pathologies in vivo^306^. In mice fed a high-fat diet, and tissues from ageing mice (25mo) with increased senescence markers, heterogeneity between models and tissues were again observed. miRNAs were also detected in the circulation of aged (25mo) compared to young (5mo) mice, suggesting potential functionality as a biomarker of ageing and senescence, though directly associating the circulating miRNAs to local senescence is needed.

miRNAs, including one associated with senescent cells (miR-31-5p), have been detected in the circulation and help predict pathology in a diagnostic setting^304,307^. In postmenopausal women with type 2 diabetes mellitus, miRNAs associated with senescence and detected in the circulation are positively associated with incident fracture risk. Further, in the urine of healthy subjects, there were clear signatures from older (57 years average) compared to younger (32 years average) patients with changes in the number of EVs and some but not all miRNAs^308^. Though not directly linked to senescence, this demonstrates the ability to detect miRNAs in the urine of patients and changes with age, the latter of which senescence plays a contributing role. Moreover, Solexa sequencing of human serum showed clear changes in miRNAs between subjects with an average age of 22-, 40-, 59, and 70 years^309^. Half of the miRNAs that changed with age had associations with senescence: miR-92a, miR-130b, miR-142, miR-375. As it is technologically possible now to detect extracellular vesicles and miRNAs in biological fluids, these approaches show potential for diagnostic assessment of senescence in the clinic. In the first instance, the contextual characterisation of EVs and miRNAs in the fluid must also be addressed in senescence-associated pathology in humans, and traced back to senescence burden in the local diseased tissue.

### Senescent cell-free DNA

Circulating cell-free DNA (cfDNA) was first identified in 1948 and later detected in the serum of cancer patients in 1977^310,311^. cfDNA possesses specificity and sensitivity to differentiate epigenetic alterations between cancerous and healthy cells^312^ making it a reliable candidate for further exploration in age-associated disorders^313^. cfDNA has been shown to be higher in older individuals compared to younger ones^314^. Increased cfDNA has been correlated with higher incidences of memory, perception, and motor skills impairment establishing a direct correlation between cfDNA and pathology of age-associated disorders^315^.

cfDNA and circulating tumour DNA (ctDNA) is being explored extensively in the field of cancer, where senescence plays multiple roles^316,317^. For example, chemotherapy-induced senescence is thought to play a role in therapy resistance and recurrence in preclinical models^318–322^. Senescent cells also have well-described epigenetic alterations; meaning it could be possible therefore, using a sensitive enough approach, to detect senescence-associated cfDNA signatures in biological fluids following therapy to assess resistance and recurrence^47,323–325^. Further, changes in cfDNA and ctDNA are becoming markers of diagnosis and responsiveness to therapy^326^. Breast cancer patients for example show increases in cfDNA compared to healthy controls^327,328^. Additionally, following three cycles of chemotherapy, the levels of cfDNA were reduced in the circulation, suggesting the potential for cfDNA to measure therapy response^329^. In 123 patients with locally advanced rectal cancer, cfDNA was collected across a 5-year period in those that had received chemotherapy (capecitabine + oxaliplatin) compared to not^330^. High cfDNA at baseline was associated with reduced time to progression and disease-free survival, suggesting their diagnostic use in stratifying risk in these patient cohorts. Similarly in a prospective study of pancreatic cancer patients, high cfDNA levels were able to predict the development of distant metastases as well as reduced overall survival^331^. The presence of the oncogenic mutation *KRAS* G12 was detected in both the tumour tissue and the cfDNA to suggest the circulating nucleic acids were potentially of tumour origin. Dynamic changes of cfDNA were detected following cytotoxic chemotherapy in castration-resistant prostate cancer patients with metastases, further demonstrating their diagnostic potential^332^. Considering the potential of cfDNA, further interrogation is required to help contribute to a panel of markers that can be utilised to detect senescent burden in liquid biopsies.

Given the abundance and heterogeneity of SASP, leveraging liquid biopsy for the detection of pertinent changes presents a promising avenue warranting further exploration. An important limitation is the technical detection limit^333^. Senescent cells, unlike rapidly dividing tumours, are low in number and so would only shed a limited number of DNA. With increased tools for low input detection in biological fluids, the feasibility of a senescent cfDNA test will improve. In the first instance, there may be opportunities where large changes in the number of senescent cells make cfDNA more easily detectable, such as before and after a senescence-inducing chemotherapy. Though there is preliminary evidence that senescent cells have unique epigenetic signatures compared to their tumourigenic counterparts, this must be characterised in more detail in a disease-specific manner^47^. In 2020, Rostami et al. successfully detected cfDNA in cell lines derived from head and neck squamous cell carcinoma (HNSCC) and non-small cell lung cancer (NSCLC). Using ionising radiation as a model, they explored the relationship between senescence and cfDNA release. Their findings revealed a delayed release of cfDNA following genotoxic stress, with ionising radiation-induced senescence negatively regulating this release^334^. The ability to isolate cfDNA from individuals ranging in age from 25 to 101, as well as from various cell lines, underscores the consistency and reliability of cfDNA detection and quantification^335^. Some studies, such as Rostami et al., demonstrated a reduction in cfDNA release using cell line models. In contrast, research by Teo et al. reported reliable detection of cfDNA across various age groups, with increased detection correlating with advancing age and deteriorating health status. Given that cfDNA release has also been linked to exosomes as a means of maintaining cellular homoeostasis, more sensitive detection methods are required to better establish its presence in different models^336^. Proteomic analysis is one method used to detect cfDNA in exosomes, which has also been employed to identify SASP proteins in extracellular vesicles (EVs). EV markers, including CD9, CD42, and CD63, have been identified using proteomic data analysis, particle size distribution, and antibody-based detection techniques^31^.

Although liquid biopsy for senescence detection is still in its early stages, further research could significantly advance precision diagnostics and treatment strategies for senescence in cancer and other age-related conditions. Using cell lines to study cfDNA release may not provide a fully reliable method due to several inherent limitations. One key issue is that cfDNA can originate from various cell types, meaning that the interpretation of results may vary depending on the tissue composition. This heterogeneity complicates the analysis and reduces the consistency of cfDNA profiling across different models. Additionally, cell line models fail to accurately recapitulate the complex in vivo microenvironment. The absence of this microenvironment can lead to differing interpretations and hinder the translation of in vitro findings to clinical settings^334^. Another significant limitation is the relatively small proportion of senescent cells present in the population. The low abundance of these cells may result in cfDNA levels that fall below detection thresholds, making it challenging to accurately measure and assess senescence-related cfDNA. This highlights the need for more sensitive detection methods and improved models that can better represent the senescent burden in various tissues, potentially improving the utility of cfDNA as a biomarker for ageing and disease. Lastly, the use of cfDNA as a sole biomarker is unlikely due to the convergence of multiple factors that lead to significant overlap between data from healthy individuals and those with various disease types. Total cfDNA levels alone offer limited clinical utility, as elevated levels are often observed in a range of conditions, making it difficult to distinguish between different disease types based solely on cfDNA quantity. However, early studies that explored the relationship between total cfDNA levels and clinicopathological data generated significant interest in the field. These foundational studies shifted the focus from total cfDNA quantity to a more detailed genetic and epigenetic characterisation of cfDNA. As a result, the genetic and epigenetic profiling of cfDNA holds great potential for improving the accuracy of diagnostics and prognostics across a wide range of diseases, further advancing the field of liquid biopsy^337^.

### Senescent cells in biological fluids

While it may be limited to tissue-specific biopsies at the moment, future research may look to using circulating cells for a faster, less invasive assessment of individualised senescence burden^338^. For example, Kalies et al. recently reported a method using combined magnetic-activated and fluorescence-activated cell sorting to aid in isolating circulating endothelial cells (CECs)^339^. The researchers found that the method enabled the isolation of sufficient populations of CECs for DNA and RNA sequencing to quantify markers of senescence. While only a small cohort was assayed (*N* = 11 per group) this is a promising step towards better clinical characterisation of an individual’s senescent cell burden.

A meta-analysis showed an association of cell cycle regulators, including those involved in senescence, with age in human blood, though this was highly context dependent^340^. For example, p16 mRNA was increased in the HIV patient leucocytes and T cells compared to healthy controls^341–343^. Senescence markers were assessed in peripheral blood mononuclear cells (PBMCs) in patients with Alzheimer’s Disease (AD), acute mild cognitive impairment (aMCI) or healthy controls^344^. SA-β-Gal activity was increased in PBMCs of aMCI patients compared to healthy controls and AD patients, whereas the proportion of PBMCs positive for p53 and p16 increased with disease aMCI and further with AD. Phosphorylated p53 and p21 were increased in peripheral blood lymphocytes in AD patients, but not those with Parkinson’s Disease (PD) or Vascular Dementia (VAD)^345^. Transcriptional increases in p21 and p16 were detected in leucocytes from patients with chronic obstructive pulmonary disorder^346^. p16 expression in peripheral T cells correlated with plasma IL-6 concentration, physical inactivity and tobacco use^347^. The expression of p16 in peripheral blood T cells of breast cancer patients before chemotherapy is associated with patients who suffered from severe fatigue post-chemotherapy^322^. Patients with Amyotrophic Lateral Sclerosis (ALS) showed an increased frequency of senescent blood lymphocytes in those with faster progressing disease^348^. A senescence signature was established for leukaemia cells following Erythropoietin treatment in vitro, a compound used to manage anaemia in the clinic^349^. This drug-induced senescence in vitro and the signature that was developed as a result correlated with reduced survival in human acute myeloid leukaemia (AML) patients.

Importantly, there are examples where expression of senescence markers in diseases were decreased compared to controls, as well as contradicting evidence depending on the method of senescence assessment used^338^. This further demonstrates the heterogeneity of the roles of senescence-associated genes in blood cell types and different diseases, which must be considered when translating into a clinical diagnostic setting to predict disease or therapy response.

### Current limitations of senescence assessment in biological fluids

The predominant limiting factor is that SASP factors are not specific to senescence^350–353^. Many of these proteins are markers of ageing and diseases such as cancer, Alzheimer’s, and cardiovascular disease. It is conceivable that senescence burden is not the sole contributor of these factors. A deep characterisation of the SASP factors present locally in a disease model before comparing them with the potential SASP factors present in the periphery will prove useful. This will identify which SASP factors are being released by the diseased tissue of interest and help understand their contribution to peripheral soluble factor signals. Some examples of this are already published. For example, in a cachexia mouse model, a senomorphic compound (SR12343) was shown to reduce SASP factors in both muscle and blood, resulting in reduced pathology^354^. In 38 patients aged 65 to 81, p16 expression in skin and adipose tissue was correlated with age and with circulating levels of VCAM1 in the bloodstream. Alternatively, p16 expression in isolated fibroblasts and MSCs correlated with secreted IL-6 and MCP-1, further highlighting the differences that require careful consideration^355^. In the aforementioned study assessing circulating SASP factors in IPF patients, circulating SASP expression was compared with expression in the lung tissue^275^. Gene expression of many assessed circulating biomarkers were also increased in IPF lungs, and was associated with increased lung P16 but not p21 expression. Further assessment of how these SASP factors change following the ablation of senescence would further strengthen the association between peripheral soluble factors and local senescence burden. Considering the heterogeneity of senescence, assessment of senescent burden by circulating factors may initially require a tissue biopsy to stratify a patient into a particular ‘SASP family’. At which point, this can guide which circulating factors in the periphery should be monitored for changes in senescence burden.

## Future directions for senescence diagnostics

Detection of senescence is of importance in diagnostic and prognostic settings due to the current preclinical evidence reviewed here. For example, in the context of cancer, senescence seems to be more highly associated with benign lesions and then lost as a cancer becomes malignant^2,163^. If there were a tool to monitor senescence in a non-invasive manner it would be feasible to identify when the ‘switch’ occurs from an early lesion, guiding clinical intervention. As this is a particular ‘early’ phenomenon in cancer, a method for identification of changes in senescence burden could promote early cancer detection, giving opportunities for improved clinical outcomes^356^. In cancer therapy, evidence of tumoural senescence following treatments has multiple potential uses. Firstly, increased intratumoral senescence following treatment could indicate the tumour has initially responded and limited proliferation. Secondly, identification of senescence burden following treatment could stratify a patient to an anti-senescence therapy, acting as a one-two punch to clear the tumour^357^. Thirdly, longitudinal monitoring of therapy-induced senescent cancer cells could also act as a marker of early cancer recurrence and senescence escape, identifying when a patient needs further treatment^318–322^.

**Fig. 2:**
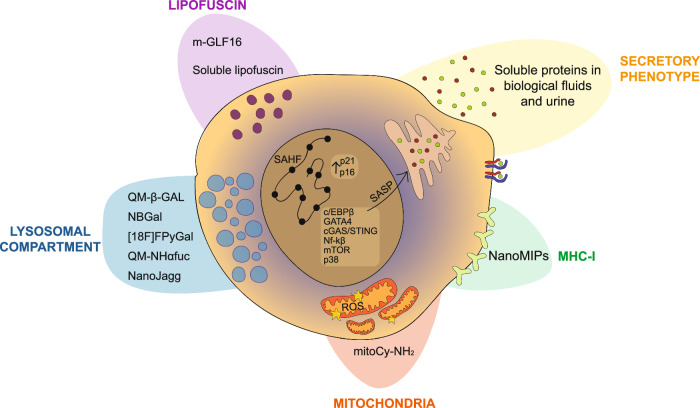
Clinically relevant diagnostic tools for senescence detection in vivo. Detection of senescence in vivo in a non-invasive way is one of the main challenges of the field. Diagnostic senoprobes exploit the increased lysosomal SA- β-Gal activity in senescence (QM- β-Gal, NBGal, [18 F]FPyGal), as well as the increased activity of the lysosomal hydrolase α-L-fucosidase (QM-Nhαfuc), or the preferential accumulation in senescent lysosomes of the probe NanoJagg. Other approaches are antibody-drug conjugates against senescent-specific cell surface markers, such as B2M, which deliver fluorescent probes, the GLF16 probe, an hydrophilic fluorescent Sudan Black B (SBB) analogue that can identify lipofuscin-positive senescent cells, and probes that detect monoamine oxidase (MAO) activity (MitoCy-NH_2_). In addition to this, proteins secreted by senescent cells as part of the SASP and also soluble lipofuscin can be detected through liquid biopsies, a cornerstone of diagnostics.

To detect the burden of human senescence in a non-invasive manner, outside of tissue biopsies for example, will lead to their increased use and translatability into the clinic. Senoprobes with evidence of live in vivo detection in preclinical models are highlighted in Fig. 2 and described in Table 2. For effective clinical usability, one could imagine that a senoprobe with multiple levels of functionality, specialised to the disease and tissue of interest would be beneficial. Combinatorial senoprobes are being developed with promising in vitro evidence, but their translation in vivo remains to be seen^267,268^. Alternative senescence markers are yet to be explored also, for example utilising SASP factors with enzymatic activity such as MMPs.

**Table 2:** Senoprobes for detection of senescence

Target	Name	Mechanism	Models tested	Advantages	Limitations	Ref.
SA-β-Gal	C12FDG	Cell-permeable fluorescent substrate that remains intracellular upon cleavage.	Living cells.	Improved resolution than conventional X-Gal.	Low tissue penetrance Autofluorescence Low cell loading Slow response rate.	^195^
	DDAOG	Far-red shifted version of C12FDG.	Living cells, tissue samples.	Improved autofluorescence and tissue penetrance.	Few evidence of effective in vivo assessment in most tissues.	^196^
	SPiDER-β-Gal	Fluorogenic substrate that allows single-cell resolution.	Living cells, tissue samples.	Improved cell permeability and retention compared with C12FDG.	Not suitable for in vivo detection.	^197^
	SG1	Two-photon imaging using an emission-based ratiometric probe.	Living cells.	Faster response than C12FDG. Photostability. Low cytotoxicity. Improved penetrance.	Limited in vivo translation.	^198^
	Gal-Pro	Emits far-red fluorescence when glycosidic bond is cleaved.	Living cells.	Rapid.Sensitive. Improved signal-to-noise.	Limited in vivo translation.	^199^
	AHGa	Naphthalimide fluorophore that emits strong fluorescence upon cleavage.	SK-Mel-103 Xenograft.	Improved tissue penetrance and in vivo transability.	Limited in vivo translation.	^200^
	HeckGal	Two-photon fluorescent probe	Ex vivo mouse kidney fibrosis and palbociclib-treated breast cancer.	Improved tissue penetration, lower phototoxicity, and minimised light scattering.	Unable to detect live in vivo activity.	^201^
	QM-β-Gal	AIEgen that, upon β-Gal activity, creates fluorescent nanoaggregates.	Living cells. Doxorubicin-treated breast cancer transplanted mice.	Rapid increase in fluorescence and persistent signal in vivo.	Requires specialised optical fibre confocal imaging for in vivo detection.	^202,203^
	GosNP	MSNs coated with a galacto-oligosaccharide cap, able to be cleaved by β-Galactosidase activity.	Yeast, human fibroblasts Cells derived from patients with Dyskeratosis Congenita.	High specificity and minimal detectable toxicity.	Unable to detect live in vivo activity.	^205^
	GalNP	Homogeneous 6‐mer galacto‐oligosaccharide coated MSNs.	In vitro and ex vivo detection of palbociclib-treated tumours and fibrotic lungs in mice.	Reduced toxicity Efficient delivery.	Unable to detect live in vivo activity.	^206^
	Nile blue loaded MSNs	Nile Blue loaded β-Gal activatable MSNs.	Palbociclib-treated mice.	Real-time in vivo senescence detection.	Limited tissue penetrance.	^207^
	Gal-(ZnPc*)_2_-NP	Disassembles with β-Gal activity to release photoactive units.	HeLa cells.	Effective detection and elimination of senescent cells.	Limited in vivo translation.	^209^
	Sulfonic-Cy7Gal	Hydrolysed by β-Gal to release Cy7 fluorescent substrate into the kidneys & urine.	Palbociclib-treated mice bearing 4T1 tumours. Naturally aged mice.	Live in vivo detection. Non-invasive. Enables longitudinal studies.	Difficult to determine local source of senescence.	^204^
	[18]FPyGal	Positive Emission Tomography tracer.	in vivo and in vitro models. Human cancer patients.	Live in vivo detection. Deep tissue penetrance.	Limited published evidence.	^215^
	[18 F]-PyGal	Positive Emission Tomography tracer.	Doxorubicin-treated mice. Aged mice. Pig knee cartilage.	Live in vivo detection in mice and pigs.	May not be senescence specific.	^216^
	[68Ga]Ga-BGal	Positive Emission Tomography tracer.	LacZ CT26 tumours or doxorubicin-treated HeLa tumours in mice.	Live in vivo detection in mice. Potential to assess signals in deeper tissues.	Signal also seen from LacZ overexpression so not senescence specific.	^212^
Sialidase	Sia-RQ	Fluorescence quenched probe that, upon cleavage, activates fluorophore.	Palbociclib-treated human hepatocarcinoma cells, Huh-7.	Long-term imaging.	Sialidase is poorly explored in senescence. Limited in vivo translation.	^224^
α-L-fucosidase	QM-NHαfuc	AIEgen esponding to α-fuc activity by fluorescence emission.	in vivo, xenograft model.	Real-time, non-invasive tracking of senescence.	Limited tissue penetrance.	^226^
Endocytosis	NanoJagg	Self-assembly of ICG dimer that accumulates in senescent lysosomes.	in vitro and ex vivo and in vivo with Palbociclib-treated SK-Mel-103 mouse xenografts.	Live in vivo detection. Deeper tissue penetrance. Potential for longitudinal monitoring.	Mechanism of senescence specificity not fully understood.	^227^
B2M	nanoMIPs	Nanoparticles loaded with DyLightTM 800 NHS Ester.	in vitro and in vivo.	Non-toxic.	May not be senescence-specific.	^230,231^
CD9	CD9-coated MSNs	MSNs loaded with a Cy5.5 fluorophore	ApoE−/− mice	Effective against atherosclerosis.	Other senescence models not explored.	^239^
Lipofuscin	SenTraGor	A lipophilic, biotin-linked Sudan Black B (SBB) analogue.	Fixed samples. Human serum Human follicular fluid.	Greater signal-to-noise ratio Chemiluminescence allows detection in biological fluids.	Not amenable to live in vivo detection.	^254,256^
	GLF16	Hydrophilic fluorescent SBB analogue.	Multiple senescent cell types.	One-step process	Not amenable to live cell detection.	^260^
	m-GLF16	GLF16 encapsulated into a copolymer micelle.	H_2_O_2_-treated human organoids. Palbociclib-treated melanoma and lung fibrosis mouse models.	Rapid and robust tracking of live senescent cells in vivo.	Fluorophore-based probe will limit tissue penetrance when translating to larger organisms for live in vivo detection.	^261^
Mitochondrial dysfunction	MitoCy-NH2 and MitoHCy-NH2	Ratiometric fluorescent probes located to the mitochondria, fluorophore released upon MAO-B activity.	BALB/c mice of different ages.	Detectable fluorescence increases with age.	A direct connection with senescence has not been proved yet.	^266^
SA-β-Gal & Formaldehyde	PGal-FA	Tandem probe requiring SA-β-Gal & Formaldehyde expression for fluorogenic response.	Bleomycin-treated lung sections. OVCAR-3 cells bleomycin-treated A549.	Enhanced selectivity in comparison with individual biomarkers.	in vivo characterisation is needed.	^267^
SA-β-Gal & Mitochondrial dysfunction	PβGal	SA-β-Gal and monoamine oxidase A (MAO-A)	Multiple senescence induction methods in different cell types.	Enhanced selectivity in comparison with individual biomarkers.	Evidence for in vivo translation is needed.	^268^

Probes for the detection of senescent cells (senoprobes) are useful in diagnostics to monitor senescent cell accumulation in pathology and in response to therapies, as well as determining the efficacy of senescence modifying treatments such as senolytics. This table summarises the myriad of senoprobes available for detection of different senescence markers, including their mode of action, models assessed, advantages and limitations of each.

*β-Gal* β-Galactosidase, *H*_*2*_*O*_*2*_ hydrogen peroxide, *MAO* monoamine oxidase, *MSN* mesoporous silica nanoparticles, *SBB* Sudan black B.

Assessment of senescence in human biological fluids is also showing promise. In the context of pathology, recent work demonstrated that it is technologically possible to track ageing signatures in human plasma with diagnostic implications^358^. This approach could be re-utilised to assess senescence burden in blood plasma, before correlating with local senescence burden in age and organ-specific disease for diagnostic purposes. Indeed, there are multiple correlative examples of senescence-associated genes in the circulation and urine that are associated with human pathology^11,20,31,272–275^. Not only this, SASP detection in the circulation can also allow monitoring of physiology, for example physical activity and calorie restriction was able to reduce circulating SASP factors^273,359,360^. There are multiple biological fluids that will be of interest for specific diseases that should be explored, including saliva, sweat, cerebrospinal fluid (CSF) and breath biopsies. In all, the development of senoprobes in the clinic has great potential and should be carried out in conjunction with senotherapeutic approaches, which will be explored in the next section of this review.

In future, our view is that rather than relying on common markers such as SA-β-Gal, P21, and P16 or looking for a novel ‘universal’ senescence biomarker, one should diagnostically identify senescence using signatures of multiple ‘markers’ according to the cell type and disease of interest. The advancement of single-cell technologies is allowing senescence characterisation at a deeper level with increasing affordability. Though it is a large undertaking, a multiomics approach to characterise senescent cells in vivo is essential for the development of a sensitive and specific senescent signature. Further, we must develop signatures that distinguish pathological senescence and physiological senescence. Physiological senescence is underexplored but required to develop new senotherapies that target senescent cells that contribute to pathology, but do not target senescent cells required for homoeostasis. In the lung for example, p16-positive cells are both present in models of fibrosis^361^ and cancer^163^, but also are required during development^57^ and to maintain a reparative niche for tissue regeneration^58^. Therefore, one should be careful when interpreting the phenotypes that result from p16 ablation in mouse models, as relying on this alone before clinical translation may limit efficacy. Instead, single-cell omics of dual models of regeneration and pathology may identify unique distinguishing features of both types of p16-positive cells. For example, in Yao et al. they tested hyperoxia during development in the mouse lung, creating a dual physiology and pathology model^57^. Single-cell spatial transcriptomics for example could potentially distinguish senescent cells that are proximal or distal to a pathological area or a regenerative area, and therefore indicate those that have a physiological or pathological function. An additional benefit of a dual model would help identify whether physiological senescent cells can become pathological in the onset of disease, or whether pathological senescent cells are a completely distinct entity.

## New senotherapies and clinical trials

**Fig. 3:**
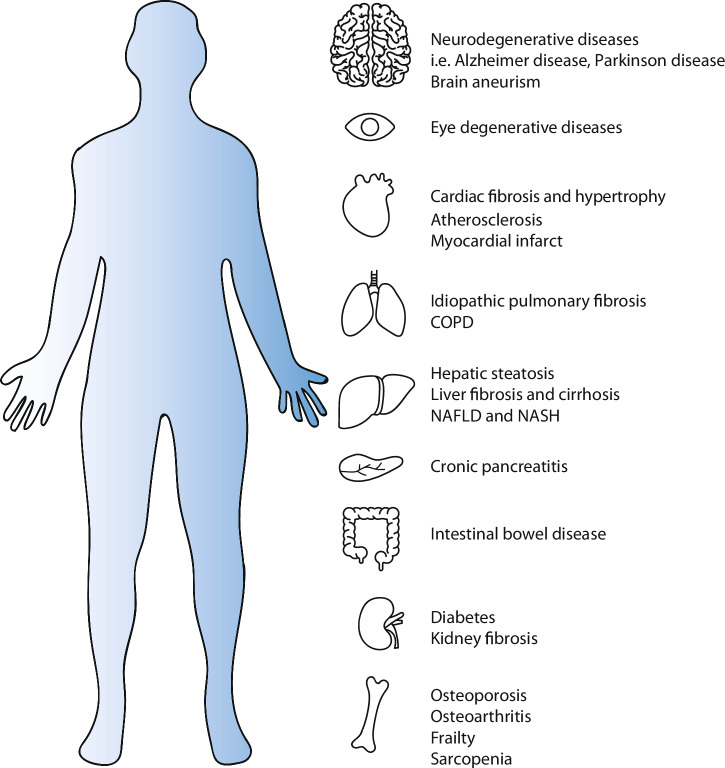
Senescence-associated pathologies beyond cancer. Accumulation of senescent cells has been linked to the onset and progression of different pathologies mainly observed during ageing^455–462^.

**Fig. 4:**
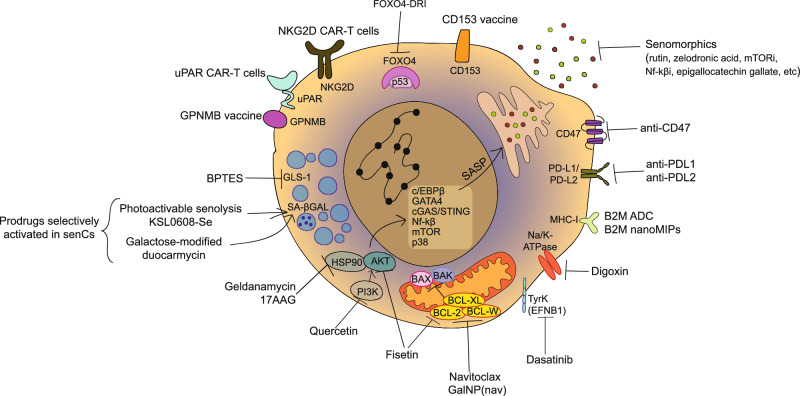
Therapeutic approaches to target senescent cells. Therapies developed to eliminate senescent cells are based on the expression of specific markers or upregulation of specific processes in the senescent cell. Compounds that target senescent cells are usually divided into senolytic, molecules that induce senescent cell death, and senomoprhics, compounds that modulate the SASP. The first-generation senolytics can interfere with anti-apoptotic/pro-survival processes active in senescent cells (Navitoclax, Geldanamycin, 17-AAG, Digoxin, BPTES, Q + D, Fisetin). New approaches leveraged the advance in nanomedicine and immunotherapy, and led to the development of nanomedicine-based and immune-based senotherapy (nanoMIPs, KSL0608-Se, galactose-modified duocarmycin, CD153 vaccine, GPNMB vaccine, uPAR CAR-T cells, NKG2D CAR-T cells, B2M ADC).

Senescence has been implicated with both the onset and the progression of different diseases, from fibrosis to cancer and neurodegenerative diseases and, more generally, with ageing (Fig. 3). Senescent cells accumulate in multiple tissues over time, and different studies have shown that the elimination of senescent cells through genetic mouse models can have beneficial effects on healthspan and lifespan. This evidence has led to an extensive research into the development of therapies that can specifically target senescent cells: these compounds are usually divided into senolytic and senomorphic drugs. Senolytics are molecules that directly induce senescent cell death, while senomorphics are compounds that can modulate the SASP in order to reduce its pathological effects (Fig. 4). The rationale underlying the development of senolytic drugs is the targeting of specific pathways or processes that are specifically active or dysregulated in senescent cells^189,362^.

## First-generation senolytics

The first senolytic drugs were discovered through the identification of possible compounds that could interfere with the “senescent cell anti-apoptotic pathways” (SCAP). Senescent cells had been previously shown to withstand high levels of damage that would normally lead to apoptotic cell death; transcriptional analysis revealed that this is due to the upregulation of anti-apoptotic/pro-survival processes, defined as SCAP^363^. Bioinformatic analyses to identify compounds that could target SCAP pathways identified 46 drugs with potential senolytic activity. Compounds meeting the criteria of being able to target multiple senescence-associated pathways, suitable for oral administration, and being natural products already approved by the FDA were chosen. This selection process resulted in the identification of dasatinib (D) and quercetin (Q) as senolytics. Dasatinib is an inhibitor of multiple kinases, including Ephrin B1 (EFNB1), ligand of Eph-related receptor tyrosine kinases. Ephrins are the largest family of tyrosine kinases, and play a crucial role in tissue and organ patterning during development; moreover, they participate in networks that inhibit apoptosis^364^. Quercetin is a flavonoid, described as an inhibitor of kinases such as PI3K and serpines, and can also target members of the BCL-2 family. As quercetin is a flavonoid, subsequent screens on flavonoids identified Fisetin as a senolytic drug^365^. Fisetin has been described as an antioxidant and anti-inflammatory agent, whose senolytic activity may depend on the downregulation of different senescence-associated pathways, such as SIRT1, BCL-2/XL, HIF1a, p53/MDM2 and AKT. It is important to note that none of these senolytic agents are universally functional, and their senolytic mechanisms of action have not been fully described. Among the SCAP, senescent cells specifically upregulate the B-cell lymphoma 2 (BCL-2) family, which is composed of three members, *BCL-2*, *BCL-XL* and *BCL-W*. Accordingly, Navitoclax, an inhibitor of the three BCL-2 anti-apoptotic proteins also known as ABT-263, was also found to be senolytic in different cell types in vitro and senescent-driven pathologies in vivo^366^. Other screening libraries led to the identification of HSP90 chaperone inhibitors (geldanamycin, 17-AAG)^367^, cardiac glycosides such as digoxin (inhibitors of the Na^+^/K^+^-ATPase)^368^ and glutaminase-1 inhibitors (BPTES)^369^ as additional senolytic drugs. An alternative approach using a FOXO4-DRI peptide was able to bind p53 and relocalise it to the cytoplasm from the nucleus, resulting in apoptosis in senescent cells^131^.

## Senomorphic compounds

Rather than directly eliminating senescent cells, another possible route to ameliorate senescence-related diseases is the modulation of the SASP through the use of senomorphic drugs. The production of the SASP is dependent on different transcription factors, such as NF-kB, mTOR and the JAK-STAT signal transduction pathways: inhibition of those factors can reduce the SASP^370^. As an example, rapamycin and other mTOR inhibitors are capable of suppressing the SASP and therefore increasing healthspan and lifespan in mice^371^. Similarly, inhibitors of NF-kB also reduce the release of SASP factors and consequently inflammation^372^. New senomorphic drugs include Rutin, an inhibitor of the interaction between ATM, HIF1ɑ and TRAF6, the bisphosphonate zoledronic acid, and epigallocatechin gallate, an anti-inflammatory and antioxidant agent extracted from green tea^373–376^. Mitochondrial dynamics have been demonstrated to play a relevant role in the modulation of SASP. Mitofusin 1 and 2 are proteins that are involved in the processes of mitochondrial fusion and morphology^377^. Inhibition of mitofusin 1 in therapy-induced senescent melanoma cells resulted in a reduction in SASP expression, particularly of the immunosuppressive factor galectin- 9^378^. This renders tumour cells more susceptible to elimination by immune cells in vivo. Another recent work has demonstrated that mitofusin 1 inhibition, targeting the mt-dsRNA/MAVS/SASP axis, can constitute an emerging effective senomorphic strategy^379^.

Apart from counteracting the inflammatory and tissue-destructive effects of the SASP, senomorphic drugs can also directly reduce the burden of senescent cells by inhibiting paracrine senescence. A main difference between senolytics and senomorphic drugs from a therapeutic perspective is the timeframe of administration of the therapy: while senolytics show efficacy with intermittent administration, treatment with senomorphics need to be continuous to maintain the suppression of the SASP. Because of this, it is believed that SASP inhibitors can lead to more side effects than senolytics.

## Efficacy of senolytics in preclinical models

The potential benefit of senolytic therapies has been investigated in a variety of preclinical animal models. The combination of quercetin and dasatinib is, as of today, the most well studied senolytic intervention in preclinical animal models. The efficacy of D + Q has been studied in Alzheimer’s disease, osteoarthritis, type 2 diabetes, heart, liver, muscle, kidney and lung disorders^361,363,380–387^.

Fisetin’s effects have primarily been studied in both progeroid and naturally ageing mouse models, demonstrating beneficial impacts on health and lifespan^388^. Furthermore, experimental models of muscular dystrophy have also exhibited positive outcomes following fisetin treatment^388^. Of note recently, Fisetin did not extend the lifespan of genetically heterogeneous mice (UM-HET3) in the Interventions Testing Program (ITP)^389^. A deeper analysis into the senescence burden with and without Fisetin in this mouse strain across the lifespan is warranted.

Navitoclax is also effective in reducing senescent burden in numerous models of disease, from neurodegenerative diseases to osteoarthritis^48,390–397^. Nevertheless, its toxicities (such as thrombocytopenia) limit clinical utility in senescence settings^398^.

HSP90 inhibitors have been tested in models of pulmonary fibrosis^367,399^, where they reduce the progression of the disease.

Senescent cells may demonstrate both deleterious and advantageous effects because of their intricate biology process. While removal of senescent cells might improve tissue function and reduce inflammation, it can also unintentionally hinder beneficial processes^400,401^. The existence of this dichotomy is a challenge in the development of senolytics which might specifically and efficiently focus on the adverse effects of senescence without causing unintentional harm.

## From research to clinical trials

in vitro and preclinical studies have yielded promising outcomes regarding the removal of senescent cells and the improvement of age-related diseases. These findings have prompted the initiation of various clinical trials, which are currently ongoing. As of today, more than 20 different clinical trials for senolytic therapies have been completed, started or are planned^189^. Most of them have been conducted in patients with highly severe diseases, such as pulmonary fibrosis, diabetic kidney disease and Alzheimer’s disease.

In 2019 the efficacy of D + Q in subjects with diabetic kidney disease was evaluated through a phase 1 pilot study. The therapy decreased senescent burden, as measured by p16 and p21 expression and SA-β-Gal activity, as well as by circulation SASP factors levels^402^.

The efficacy of D + Q was also evaluated in idiopathic pulmonary fibrosis (IPF) patients, demonstrating good tolerability and a reduction in physical dysfunction associated with IPF^403^. These promising results suggest the need for further assessment of D + Q in larger randomised controlled trials, to definitively evaluate whether the positive outcome is due to a decrease in senescence burden.

A recent publication highlighted a human trial using senolytic therapy to modulate Alzheimer’s Disease progression^404^. A 3-month intermittent D + Q regimen that was well tolerated in the patients resulted in clear enrichment for both compounds in the blood, but only Dasatinib in the CSF of patients. Cognitive and neuroimaging endpoints did not alter from the treatment and there were very limited changes in SASP and disease biomarkers in the plasma or CSF, though they were underpowered for robust assessment of these outcomes. The tolerability demonstrates the potential for expanding the trial and perhaps altering quercetin to improve bioavailability in CSF.

In a phase 2 randomised control trial of 60 postmenopausal women, intermittent D&Q therapy across 20-weeks was used to assess the efficacy of senolytics in preventing age-related bone loss^405^. No serious adverse events were described in the senolytic cohort, demonstrating safety, however, no changes were observed in bone resorption, the primary clinical endpoint. At 2- and 4- weeks there was a statistically significant increase in a marker for bone formation, a secondary endpoint. A post-hoc analysis showed that women in the highest tertile for T-cell *P16* mRNA levels showed a different response to others, demonstrating both increases in the bone formation marker, decreases in the bone resorption marker and improved bone mineral density in the radius. This demonstrates the importance of stratifying individuals for senescence burden to identify individuals where senolytics may prove most useful clinically.

In the case of osteoarthritis, the use of UBX0101 (nutlin-3a) has been interrupted due to the increase in pain in the patients affected by the disease. A BCL-xl inhibitor, UBX1325 (foselutoclax) was used in a phase 1 trial for patients with diabetic macular oedema^406^. A single intravitreal injection of this compound did not show dose-limiting toxicities, and improved visual acuity in 5 of 8 patients at the latest time point assessed, 24 weeks later.

Although senolytic treatments have demonstrated effectiveness in animal models, the process for translating these discoveries to humans has proven to be difficult. Due to the complexity of the human body, there is considerable diversity in how individuals react to different treatments^407^. Research has demonstrated that removing senescent cells exhibits a considerable degree of efficacy in alleviating the burden they impose. Nevertheless, the extent of these advantages demonstrates significant variability across individuals. Evaluating the precise impact of senolytics in vivo is a formidable undertaking currently because of the complexity in identifying biomarkers of senescence^20^. One initial approach could be to stratify patients according to their senescence burden before placing those with higher senescence levels on a senotherapeutic regimen.

## Nanomedicine-based senotherapy

As discussed in the context of senoprobes, nanomedicine has improved many aspects of classical senolytics drugs, helping to achieve the goal of precise and safe senolysis. With the technology of nanoparticles and prodrugs that take advantage, in many cases, of the overabundance of β-galactosidase in senescent cells, scientists have developed adaptable devices that increase bioavailability, stability and in consequence decrease the on- and off-target toxicity of widely employed senolytics^408,409^. Significant progress has been made recently in this field that will likely impact clinical translation.

Galacto-coated senolytic nanoparticles such as GalNP(nav) have shown in vivo a reduction in tumour volume in tumour xenografts treated with palbociclib, as well as decrease navitoclax-associated thrombocytopenia^206^. The same effect was reported in a human lung cancer xenograft model, in which the sequential administration of a senescence-inducing drug in vivo, cisplatin, and the galacto-encapsulated nanoparticle with navitoclax had a synergistic impact on the anti-tumour efficacy^410^. The combination of the sequential delivery in nanoparticles of a senogenic drug and a senolytic was tested in breast cancer cells with improved therapeutic impact than classical approaches based on nanoparticle stigmergy communication^411^.

Galactose-modified duocarmycin that functions as a prodrug presented senolytic activity in vivo in a mouse whole-body irradiation model as well as in a mouse model of adamantinomatous craniopharyngioma^412^. Recently a nanodevice was designed to target matrix metalloproteinase 3, an enzyme that is secreted in high levels by irradiated senescent fibroblasts. By loading nanoparticles with navitoclax coated with a substrate for this enzyme, the authors demonstrated a specific senolytic effect in vitro^413^.

Molecularly imprinted nanoparticles (nanoMIPs) against a surface protein in senescent cells, B2M, were mentioned earlier as able to detect senescent cells in a live in vivo setting with relative safety in mice. These have also shown senolytic efficacy by loading the nanoMIPs with the senolytic dasatinib^230^. Sphingomyelin nanosystems adorned with a peptide that target surface receptor CD47 expressed in senescent cells represent a novel intervention with senolytic activity^414^.

Another promising strategy is the design of antibody-drug conjugates, which take advantage of the expression of molecules exclusively on the membrane surface of senescent cells, in order to deliver the cytotoxic agent only in the senescent population. This approach improves the safety and selectivity of already known drugs that have been excluded from further validation due to their high cytotoxicity and the absence of selectivity. Strategies based on this rationale are the beta-2-microglobulin (B2M) Antibody-Drug Conjugate (ADC), whose efficacy has been tested in vitro in colon and bladder cancer cell lines, and the apolipoprotein D ADC tested in senescent skin fibroblasts both in vitro and in vivo^231^.

A groundbreaking approach represents photodynamic therapy that relies on nanocarriers loaded with photosensitive drugs that accumulate after light irradiation exposure in tumour cells inducing photo-cytotoxicity. This can be selectively translated to senescent cells incorporating a β-galactosidase substrate inducing precise senolysis that has demonstrated promising results^209,415^.

## Immune-based senotherapy

The identification of senolytic drugs has been mainly based on screening compound libraries that target processes which are specifically active in senescent cells. Senolytic clinical trial evidence discussed prior suggests that clinical translation of these compounds has shown some but limited efficacy. To avoid senolytic toxicities, new approaches for the elimination of senescent cells have been explored based on the interaction of senescent cells with the immune system.

It is widely known that senescent cells can recruit different immune populations through the SASP^416,417^. While the presence of senescent cells is beneficial in the short term to promote tissue regeneration and repair^70,418–420^, the persistence of senescent cells can have detrimental effects due to their pro-inflammatory phenotype^5,370^. Therefore, clearance of senescent cells is needed to maintain tissue homoeostasis. Immune cells are responsible for senescence clearance physiologically, and the decreased activity of the immune system during ageing has been linked to the increased accumulation of senescent cells^421^. Both the innate and the adaptive branches of the immune system are involved in the elimination of senescent cells, such as macrophages, NK cells and T cells^417,422,423^. Modulating the immunogenic and immunosuppressive properties of senescent cells and consequently their crosstalk with the immune system holds promise for senotherapeutic interventions.

One strategy is modulating signals from senescent cells that negatively regulate the activity of the immune system. It has been described that senescent cells, both in cancer, ageing and age-related diseases, upregulate the expression of the so-called “don’t eat me signals”, PD-L1^424^ and PD-L2 and CD47^318,425,426^. PD-1/PD-L1 and PD-L2 are one of the most studied signals that inhibit the interaction between T cells and the target cell, while CD47 is responsible for the inactivation of phagocytosis in macrophages. These pathways have been largely studied in the context of cancer, and molecules that target these interactions have been developed and studied also in the context of senescence, with promising results that will need further validation.

Recently, a lot of attention has focused on the development of Chimeric Antigen Receptor T-cell (CAR-T) cells against senescence. In 2020, Amor and colleagues identified urokinase plasminogen activator surface receptor (uPAR) as a specific Surface membrane target of senescent cells and designed uPAR-targeted CAR-T cells, which efficiently remove senescent cells in both in vitro and in vivo models of liver fibrosis and cancer^427^. Apart from ameliorating senescence-associated pathologies in young mice, the same CAR-T cells have shown beneficial effects also in aged mice, improving both the exercise capacity and the metabolic dysfunction^428^. Moreover, the CAR-T cells also present a prophylactic activity, and are capable of long-lasting effects after a single dose administration^428^. These findings underscore the promising clinical prospects of employing senolytic CAR-T-cell therapy in addressing chronic conditions. Recently, a new study has shed light on the existence of uPAR+ senescent cells within the aged intestine; removal of these senescent cells through CAR-T cells therapy enhances epithelial integrity and intestinal homoeostasis while fostering the regenerative capacity of intestinal stem cells^429^.

In addition to uPAR, another target used to develop CAR-T cells against senescent cells is the NKG2D ligands. NKG2D ligands are upregulated in senescent cells both in vitro and in vivo, independently of the stimuli that have been used to induce senescence. NKG2D CAR-T cells selectively kill senescent cells in vitro and improve the multiple ageing-associated pathologies in aged mice. Moreover, they have shown promise in delaying the ageing phenotype in irradiated mice. In aged non-human primates, autologous T cells equipped with the NKG2D CAR have been observed to delay the onset of senescent cell accumulation in tissues^430^.

A further strategy to target and eliminate senescent cells in vivo is the development of vaccine-based approaches. The first work describing a potential vaccine against senescent cells was published in 2020 by the lab of Hiromi Rakugi. In particular, the group identified senescent T cells, defined as CD4^+^ CD44^high^ CD62L^low^ PD-1^+^ CD153^+^ cells, in the visceral adipose tissue of obese individuals which were targeted using a CD153 vaccination in mice fed with a high-fat diet. Vaccination with a CD153 peptide was effective in reducing senescent T-cell accumulation and improved glucose tolerance and insulin resistance^431^. Although effective, this vaccine could only affect a subset of senescent cells, the senescent T cells, due to their upregulation of the expression of CD153. However, this approach of targeting disease-specific senescence markers is a promising first step in reducing pathological senescence in vivo.

The identification of an antigen that is truly specific to senescence has continued to pose a significant challenge. As mentioned in the previous section, Suda and colleagues identified the transmembrane protein glycoprotein nonmetastatic melanoma protein B (GPNMB) as a putative senescence-specific antigen through a transcriptome-based approach. After validation of the expression of this protein on senescent vascular endothelial cells and in atherosclerotic patient samples, the group designed peptides that corresponded to the extracellular domain of the protein, with the aim of implementing a vaccination-based approach to target GPNMB-positive cells. Notably, vaccination with GPNMB reduced senescent cell burden in mice fed with a high-fat diet, and it also ameliorated metabolic parameters. Moreover, the same vaccine was also able to reduce atherosclerotic plaque burden in an atherosclerosis mouse model. In a mouse model of Hutchinson-Gilford progeria syndrome, the GPNMB vaccine increased the median lifespan from 21 to 25 weeks^172^. Thus, this study illustrates that utilising vaccines to eradicate senescent cells offers potential in treating cardio-metabolic conditions, along with other vascular and age-related characteristics.

A recent study demonstrated that inhibition of sodium–glucose co-transporter 2 (SGLT2) induced senolysis^432^. Canaglifozen, an SGLT2 inhibitor, was able to reduce the senescence burden in visceral adipose tissue, inflammation and metabolic dysfunction within 7 days in mice on a high-fat diet. Canaglifozen increased PD-L1 expression in senescent cells, indicating the senolytic effect may be through increased immune clearance. Indeed, neutralising CD3 (T-cell) antibody treatment in Canagliflozin-treated mice stopped the senescence clearance seen. In ApoE-KO mice fed a western diet to model atherosclerosis, lifespan was increased in both males and females following Canaglifozen treatment. Reduced aortic SA-β-Gal expression and Oil Red O staining were observed, indicating reduced atherosclerosis. Leveraging the immune system and its interaction with senescent cells represents a novel and potent strategy for clearing senescent cells and improving ageing-associated diseases.

## Innovative and context-specific senolysis

To improve translation of senolytics to the alternative innovative approaches to senolysis are being developed. Conventional systemic senotherapy administration can fail to reach or improve local tissue conditions, leading to the need for higher doses and resulting in significant toxicity. A tissue-targeted drug distribution mechanism can specifically reach affected tissues without causing systemic cytotoxic effects. Therefore, there is a need for novel innovative senolytic strategies.

In naturally aged mouse models, two research teams have developed liposomes that target white adipose tissue or bone specifically, loaded with senolytics to ameliorate age-related hepatic steatosis and senile osteoporosis, respectively^433,434^. In the first case, researchers discovered that age-associated hepatic steatosis was due to the release of free fatty acids from senescent white adipose tissue. Standard systemic administration of senolytics reduces lipid accumulation in hepatocytes but does not eliminate them and can exacerbate liver disease progression, rendering senotherapy ineffective. This prompted the authors to explore tissue-targeted senolysis. The delivery of D&Q in liposomes to white adipose tissue improved age-related liver steatosis and significantly decreased free fatty acid levels. Treating osteoporosis locally poses a challenge due to the dense bone structure and limited blood supply. This motivated the authors to design bone-targeted liposomes that deliver quercetin. This targeted treatment stimulated bone formation and prevented degradation in age-related osteoporosis, improving conventional quercetin administration. Local delivery by intramyocardial injection of ABT-263 loaded in poly(lactic-co-glycolic acid) nanoparticles in a rat model of myocardial ischaemia-reperfusion restored impaired cardiac function without toxic effects^435^. These promising results shed light on the potential for therapeutic translation of senolytic administration in patients experiencing acute myocardial infarction via intracoronary infusion, minimising the risk of systemic toxicity. These examples demonstrate where context-specific senolysis could prove advantageous for targeting specific senescence-associated diseases.

An innovative senolytic approach has been utilised by taking advantage of the senescent hypersecretory phenotype^10,436^. Adaptations to their secretory function involve structural changes at the Golgi level^437–439^. These changes allow the coupling of the production and secretion needs of the factors belonging to the SASP and cause the coatomer protein complex I (COPI) protein-dependent pathways involved in vesicular trafficking to undergo hypertrophy. McHugh et al. showed that this vulnerability can be exploited as a senolytic therapy. Specifically, it was observed that by interfering with COPI expression, proteins accumulate intracellularly in both proliferative and senescent cells, but apoptosis was only observed in the latter. This was thought to be by triggering a proteotoxic response. In in vivo assays, the intervention of this pathway effectively eliminated senescent cells by preventing the propagation of pro-tumorigenic senescent signalling in murine xenograft models. Additionally, a delay in lung parenchymal fibrosis was observed in those senescent cells with decreased secretory capacity when COPI expression was modified. This non-conventional mechanism of senolysis unveils new avenues for exploration across various diseases^440^.

Recently, a preprint by Kureel et al. have claimed that senescence can be reversed using a mechanical approach involving physical pressure waves of low-frequency ultrasound (LFU)^441^. The authors assessed multiple senescence induction methods and demonstrated heterogeneous findings. Senescent Bleomycin-treated Vero cells showed an increase in cell growth and motility after two pulses of 30 min LFU over 24 h. Sodium butyrate-induced senescent Vero cells subjected to LFU demonstrated reduced p53, γH2AX, and H3K9me3 signals and decreased ROS production. In late passages of human foreskin senescent cells, the authors showed a decrease in secreted SASP factors and an increase in telomere length, and the absence of apoptosis. Mechanistically, it was proposed that LFU involves an increase in calcium influx and autophagy, as evidenced by the synergistic effect of the autophagy inducer rapamycin on growth stimulation by LFU. The authors highlighted the advantages of this biophysical therapy over biochemical-based interventions. This technology is non-invasive due to the easy penetration of ultrasound into human tissue. Before this data is extrapolated further, efficacy outside of the two cell lines must be assessed over a longer period of time. Although it is claimed that the biophysical action is independent of the tissue, this should be considered because of the heterogeneous nature of senescent cells that may require optimisation of the parameters of frequency, power, and duty cycle^442^. When assessing the potential reversal of senescence, one must consider which features are reversed or not, as oncogenic senescent cells re-entering the cell cycle is a tumorigenic process. More research is needed to answer these questions and move to in vivo models, which would be a valuable tool for studying the microenvironment interactions and the impact of LFU therapy.

## Discovery of senolytics through machine learning approaches

With the advancement of artificial intelligence (AI) technology, the use of machine learning to discover senolytics is being established. Recent work has demonstrated that drug repurposing can be efficiently achieved based on machine learning algorithms training with 58 senolytic compounds^443^. After validation tests, ginkgetin, periplocin and oleandrin were identified as robust senolytic compounds. The search for a drug with the senolytic signature of dasatinib led to the discovery based on gene expression profiles of substitutes by a computational identification^444^. These cost-effective approaches can minimise the experimental times and ensure the utilisation of literature data. Morphological changes related to the transition to the senescence state are part of an outstanding and widely established feature of senescent cells^445^. Based on these characteristics, a convolutional neural network (CNN) was trained with phase-contrast images of senescent endothelial cells to identify them^446^. A scoring system named Deep-Sesmo based on this CNN could successfully identify four senolytic compounds that act through the inhibition of the inflammatory response using a kinase inhibitor library. Analysing nuclear morphology characteristics of senescent cells, machine learning classifiers could successfully distinguish a senescent population from cells undergoing quiescence or DNA damage^447^. The algorithm was also used to identify drugs that induce senescence preferentially in cancer or non-cancerous cells. The team developed a tissue senescence score that was able to estimate levels of senescence in vivo in tissue sections of a model of liver pre-neoplasia. Moreover, senolytic activity could also be evaluated in vivo. The algorithm also could detect senescent cells in human samples from patients with nonalcoholic fatty liver disease. Together, AI and machine learning will likely increase the pace of senolytic discovery and translation in the coming years^448^.

## Future perspectives of senotherapy

Senescence plays a dual role in pathophysiology, contingent on the duration of its presence in the tissue of interest. The transient presence of senescent cells in tissues yields beneficial effects in development, physiology, and pathological contexts, where it facilitates restoration of tissue homoeostasis^449^. However, senescence persistence is responsible for its detrimental consequences, mainly attributed to the continuous exposure to the SASP^11,450^. Beyond the efficacy in neutralising these cells, concerns regarding side effects and toxicity from systemic administration have prompted a shift towards more precise therapeutic strategies. These include immune-targeted senescent-specific antigens, nanocarriers loaded with photosensitising prodrugs, and approaches tailored to specific tissues. These advancements ensure the administration of low doses, minimising the risk of toxic effects^362^.

The discovery of novel senolytics would benefit from senolysis screening of in vivo-derived senescent cells. Recently, a novel screening platform for identifying senolytic compounds was developed using isolated cells from a mouse model with an induced senescence mouse model^399^. In this work, the authors utilise the ultrasensitive fluorescent reporter of the p16 (INKBRITE) mouse model. Senescence is induced, and cells derived from specific tissues are isolated using FACS based on GFP staining. GFP+ (p16+) and GFP- (p16-) cells are mixed and plated together. Through high-throughput screening of senolytic compounds that selectively eliminate GFP+ cells, combined with precision-cut slices of selected tissue screening ex vivo of candidates and in vivo validation, the authors establish a pioneering screening platform that employs in vivo-generated senescent cells. Approaches for screening senolytic efficacy of in vivo-generated senescence, also possible with the p21:GFP zebrafish reporter^68^, will likely lead to more specific senolysis approaches.

## Conclusions

The widely established heterogeneity of senescent cells represents a major challenge in senescence research. This characteristic involves not only changes associated with the kinetics of the onset of the senescence programme but also variations based on the trigger, tissue, and sex. Despite technological advances, the basic mechanisms that govern ageing and senescence are not completely understood. Alternative emerging animal models such as zebrafish have been developed to study these processes, understand the consequences of senescence manipulation, and complement mammalian systems. The search for the ideal biomarker, or combinations thereof, is still ongoing. Many advances have been made in finding a measurement-stable biomarker detectable in peripheral tissues and correlated with local senescence and disease, though further evidence is needed. Additionally, the identification of a reliable biomarker of senolysis in vivo for tracking drug efficacy in individuals is key. Self-collected samples, like saliva and urine for human research translation, should be investigated further.

Senotherapy research that uses a conventional approach relying on systemic administration to cure or delay tissue-specific diseases faces numerous challenges. Context-specific senolytic tests should address this issue by concentrating on evaluating compounds in an ex vivo, tissue-specific manner, thus avoiding off-target toxicity effects^16^. Another underexplored topic is sex differences in senescence^451^. Reports indicate differential effects of senolytics in male and female preclinical models^18,452,453^. Additionally, the immune system ages differently according to gender^454^, which may cause differences in the effectiveness of immune-based senotherapy that are not completely understood. The field should evolve by examining these differences and integrating the data into clinical trial studies.

**Fig. 5:**
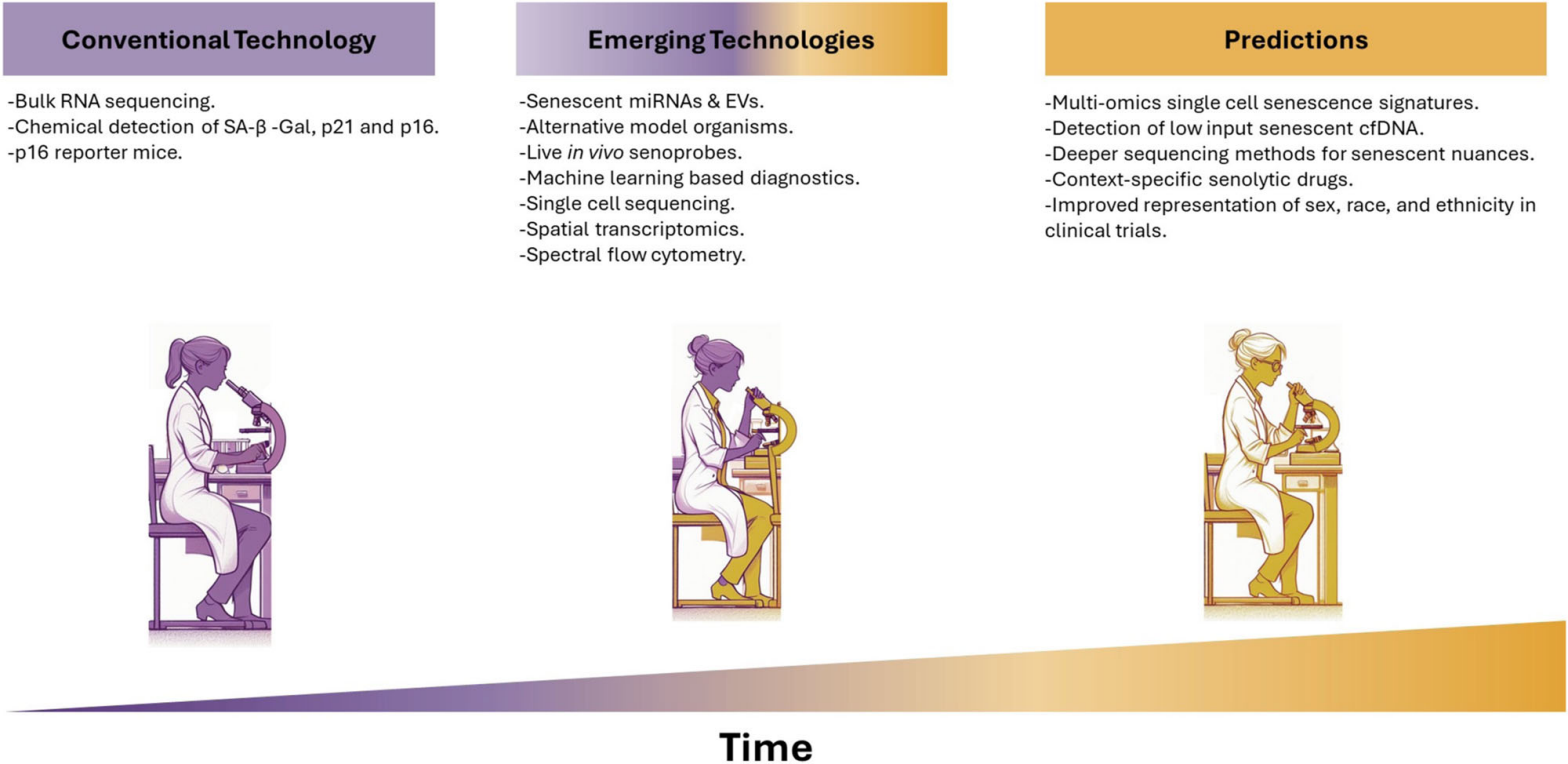
Framing the senescence research pathway: conventional, emerging, and future technologies. Over the years, senescence research has evolved, leading to the development of sophisticated techniques, biomarkers, and biosensors for the accurate characterisation, labelling, and neutralisation of senescent cells. The progression from conventional methodologies to emerging technologies in the field is illustrated. Additionally, predictions for how the field will continue to evolve are explored, highlighting the potential for even more sensitive and effective techniques in the future. EVs Extracellular vesicles, cfDNA cell-free Deoxyribonucleic Acid, miRNA micro RNA.

Our framework for senescence research of past, present and recommendations for the future is summarised in Fig. 5.
